# Nicotinamide Mononucleotide (NMN) Works in Type 2 Diabetes through Unexpected Effects in Adipose Tissue, Not by Mitochondrial Biogenesis

**DOI:** 10.3390/ijms25052594

**Published:** 2024-02-23

**Authors:** Roua Gabriela Popescu, Anca Dinischiotu, Teodoru Soare, Ene Vlase, George Cătălin Marinescu

**Affiliations:** 1 Department of Biochemistry and Molecular Biology, Faculty of Biology, University of Bucharest, 050095 Bucharest, Romania; 2 Independent Research Association, 012416 Bucharest, Romania; 3 Blue Screen SRL, 012416 Bucharest, Romania; 4 Pathology Department, Faculty of Veterinary Medicine, University of Agronomic Sciences and Veterinary Medicine of Bucharest, 050097 Bucharest, Romania; 5 Animals Facility Laboratory, Cantacuzino National Institute for Medico-Military Research and Development, 013821 Bucharest, Romania

**Keywords:** nicotinamide mononucleotide (NMN) effects, NMN proteomics, DIA SWATH proteomics, protein interaction network, pathway analysis, NMN type 2 diabetes

## Abstract

Nicotinamide mononucleotide (NMN) has emerged as a promising therapeutic intervention for age-related disorders, including type 2 diabetes. In this study, we confirmed the previously observed effects of NMN treatment on glucose uptake and investigated its underlying mechanisms in various tissues and cell lines. Through the most comprehensive proteomic analysis to date, we discovered a series of novel organ-specific effects responsible for glucose uptake as measured by the IPGTT: adipose tissue growing (suggested by increased protein synthesis and degradation and mTOR proliferation signaling upregulation). Notably, we observed the upregulation of thermogenic UCP1, promoting enhanced glucose conversion to heat in intermuscular adipose tissue while showing a surprising repressive effect on mitochondrial biogenesis in muscle and the brain. Additionally, liver and muscle cells displayed a unique response, characterized by spliceosome downregulation and concurrent upregulation of chaperones, proteasomes, and ribosomes, leading to mildly impaired and energy-inefficient protein synthesis machinery. Furthermore, our findings revealed remarkable metabolic rewiring in the brain. This involved increased production of ketone bodies, downregulation of mitochondrial OXPHOS and TCA cycle components, as well as the induction of well-known fasting-associated effects. Collectively, our data elucidate the multifaceted nature of NMN action, highlighting its organ-specific effects and their role in improving glucose uptake. These findings deepen our understanding of NMN’s therapeutic potential and pave the way for novel strategies in managing metabolic disorders.

## 1. Introduction

The incidence of type 2 diabetes mellitus (T2DM) has been steadily rising on a global scale. The International Diabetes Federation (IDF) estimates that the number of adults living with diabetes has quadrupled since 2000. In 2021, it was reported that approximately 537 million adults had diabetes, and this number is projected to rise to 783 million by 2045 if current trends continue [1]. Factors contributing to the increased incidence include lack of physical exercise, unhealthy dietary patterns, obesity, population aging, and urbanization [2,3,4]. The rise in T2DM cases presents a major public health challenge, necessitating concerted efforts in prevention, early detection, and effective management strategies to mitigate its impact on individuals and healthcare systems worldwide. The treatment of diabetes has become an increasingly expensive burden on global healthcare systems, with worldwide expenditure reaching USD 966 billion in 2021, according to the International Diabetes Federation [1], representing a significant increase of 32.8% compared to the USD 727 billion spent in 2017 [5], underscoring the urgent need for innovative treatments and interventions.

Glucose metabolism is a fundamental process, vital for energy homeostasis in mammals, orchestrated through a complex interplay among various organs and tissues. Among these, the liver, muscle, adipose tissue, and brain stand as the primary consuming and target tissues for glucose utilization [6]. The liver regulates blood glucose levels, while muscle tissue plays a significant role in glucose uptake and energy expenditure [7,8]. Adipose tissue, traditionally viewed as an energy reservoir, is increasingly recognized for its active involvement in glucose metabolism [9]. The brain, a glucose-dependent organ, demands a continuous and tightly regulated supply of glucose to support its functions [10]. Understanding the intricate correlations between these vital organs is crucial for unravelling the complexities of glucose homeostasis, metabolic health, and related disorders, like T2DM.

Previous studies have reported that β-nicotinamide mononucleotide (NMN) as a precursor of nicotinamide adenine dinucleotide (NAD^+^) ameliorates several age-related diseases, including T2DM, characterized by high blood glucose levels and insulin resistance [11,12,13]. Besides increasing the intracellular levels of NAD^+^, which plays a vital role in regulating the cellular redox state, NMN acts through several biological and chemical processes, most notably being DNA repair, energy metabolism, and stress response [14]. Previously, it was proposed that raised NAD^+^ levels activate SIRT1, leading to mitochondrial biogenesis by upregulating the expression of transcription factor A mitochondrial (TFAM) and peroxisome proliferator-activated receptor gamma coactivator 1-alpha (PGC-1α) as well as increased DNA repair and AMP-activated protein kinase (AMPK) activation [12,15,16]. The efficiency of NMN in the treatment of diet and age-induced diabetes in mice was previously reported [11,17].

However, human trials results are disappointing [18], and the previously proposed NMN-induced mitochondrial biogenesis idea is doubtful. Previous mechanistic approaches failed to provide a significant response to the question: If NMN works in T2DM, where is glucose going? The main flaws identified in these studies were most probably unreliable transcriptomics data and unreliable Western blots (for example, choosing actin as a reference protein, which our data show is dramatically affected by NMN treatment (Figure S12)).

In metabolic and signaling pathways, enzymes (that are proteins), play the effector role in chemical reactions, contributing to cellular maintenance and allowing for rapid adaptation to environmental changes. Quantifying their expression levels might provide novel insights into the intricate connections between diverse metabolic and signaling pathways [19]. Considering these, our study represents an effort to see the big picture, a proteome-centric approach to elucidating the underlying mechanisms and the therapeutic potential of NMN in the treatment of T2DM through the analysis of its proteome-level effects, including energy metabolism, in an in vivo diabetic mouse model as well as in two in vitro models (C2C12-derived myotubes and HepG2 cells, respectively). This investigation used state-of-the-art data-independent acquisition (DIA) technologies in mass spectrometry, specifically SWATH-MS, as well as label-free and library-free peptide mapping using neural networks and interference correction to achieve deep proteome coverage. An integrated map of the molecular mechanisms underlying the effects of NMN in T2DM treatment is provided.

## 2. Results

The differential expression changes in the proteome of an in vivo T2DM mouse model as well as in in vitro models of hyperglycemic muscle (myotubes derived from C2C12 cells) and liver (HepG2 cells) have been explored. A thorough library-free and label-free proteomic analysis has been conducted. The spectra library was created from actual DIA data coupled with fasta files for the *Mus musculus* and *Homo sapiens* proteomes, respectively. Overall, we quantified a total of 1728 unique proteins and 20857 precursors in mouse muscle tissue at a false discovery rate (FDR) < 1%. In mouse liver, we identified 3097 unique proteins and 31,501 precursors, while in mouse adipose tissue, we found 3217 unique proteins and 35,516 precursors. In addition, we quantified 3814 unique proteins and 43,320 precursors in mouse brain and 3855 unique proteins and 35,481 precursors in myotubes derived from C2C12 cells. Finally, in HepG2 cells, we detected 3541 unique proteins and 43,565 precursors across five biological replicates, each multiplied by three technical replicates. The effect of NMN treatment was evaluated by highlighting significant changes in protein expression levels. The Limma test was performed in PolySTest [20], which found significant changes (*p* value < 5%) in 252 proteins in mouse muscle tissue, 337 proteins in mouse liver, 411 proteins in mouse adipose tissue, 120 proteins in mouse brain, 1200 proteins in C2C12 myotubes, and 984 proteins in HepG2 liver cells. All significantly changed proteins are available in Supplemental Information (Tables S1–S6) and were used as input for pathway enrichment analysis.

The PathfindR module in the R statistical software package was used to construct the protein–protein interaction network (PIN) for *Mus musculus* and *Homo sapiens* from the STRING proteins database. Using PathfindR (version 1.6.4), an enrichment chart containing the top 20 affected pathways and a term–gene graph containing the top 10 pathways were generated. The KEGG pathways [21,22,23] were generated by the Pathview R module (version 1.38.0) and show the relative expression of each protein from the NMN-treated condition compared to the untreated condition.

### 2.1. NMN Significantly Improved Glucose Uptake from Bloodstream in HFD Mice, But Not through Insulin Resistance Effects in Muscle or Liver

**Figure 1:**
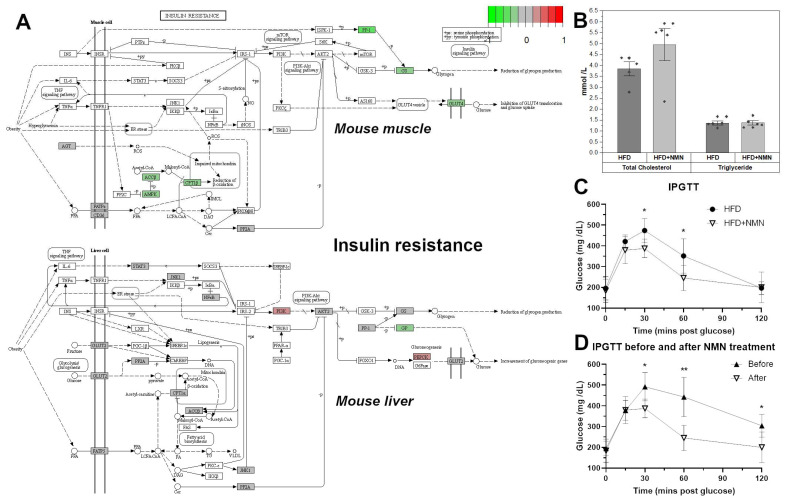
C57BL/6J mice develop severe glucose uptake deficiency on high-fat diet (HFD). (**A**) NMN treatment effects on the KEGG insulin resistance pathway in mouse muscle and liver. The color of the boxes represents the log2 fold change of the protein abundances, represented for HFD + NMN group versus HFD group. Red: upregulated; green: downregulated; grey: no significant expression change. GS and GLUT4 are significantly downregulated in muscle tissue of treated mice. (**B**) Serum total cholesterol and triglycerides in HFD and HFD + NMN-treated mice, 7 days after treatment. (**C**) intraperitoneal glucose tolerance test (IPGTT) in HFD and HFD + NMN-treated mice after 7 days. (**D**) IPGTT in HFD + NMN-treated mice before and after NMN treatment. The data are illustrated as average values of the groups (*n* = 5) ± standard deviation of the mean (STDEV) and statistical significance between HFD and HFD + NMN groups. * *p* < 0.05; ** *p* < 0.01.

No significant differences in the serum total cholesterol and triglyceride levels between the HFD group and the HFD + NMN group were noticed (Figure 1B). The HFD + NMN group had higher levels of total cholesterol (4.954 mmol/L) compared to the HFD group (3.848 mmol/L). Similarly, the triglyceride levels were slightly higher in the HFD + NMN group (1.378 mmol/L) compared to the HFD group (1.364 mmol/L); however, the differences were not statistically significant (*p* = 0.14) (*p* = 0.11). The results of the IPGTT test indicate differences in glucose absorption patterns between the HFD group and the HFD + NMN-treated group (Figure 1C). After NMN treatment (Figure 1D), the HFD + NMN group had significantly lower glucose levels compared to the HFD group 15, 30, 60, and 120 min after an intraperitoneal injection of glucose. Surprisingly, the GLUT4 glucose receptor was significantly downregulated in the muscle tissue of HFD + NMN treated mice, as well as glycogen synthase (GS).

### 2.2. Protein Synthesis Is Mildly Impaired by NMN through Spliceosome Downregulation in HFD Mouse Liver

**Figure 2:**
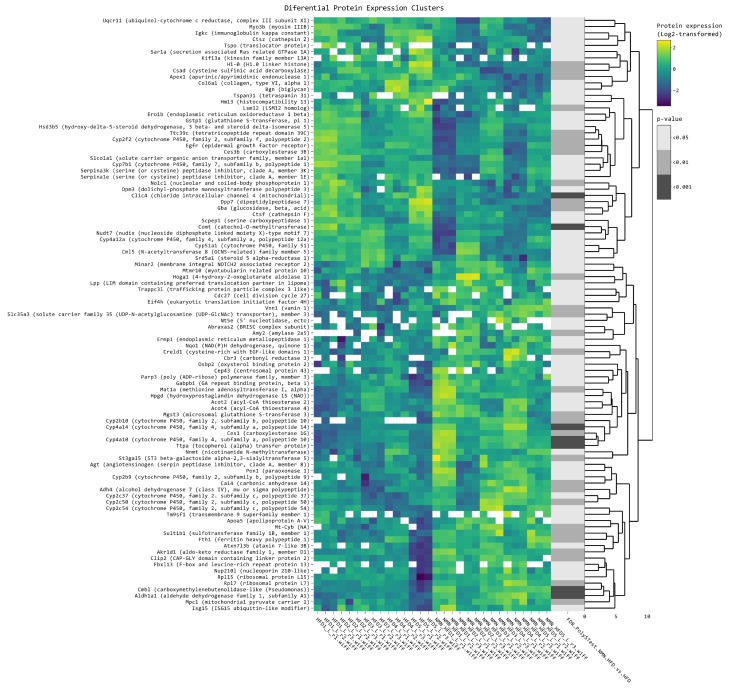
Clustered heatmap of the differentially expressed proteins in mouse liver. Clustered heatmap of the 103 differentially expressed proteins in mouse liver tissue, filtered with log_2_FC threshold set to exclude the interval −0.3, 0.3, *p* < 0.05. Yellow color represents upregulation, while blue represents downregulation. From left to right, expression values (log_2_ transformed) for replicates (5 biological × 3 technical) are shown for the HFD group and for the HFD + NMN-treated group, followed by significance values of comparison to HFD group.

In mouse liver samples, 103 proteins (Table S1, Figure 2) showed a significant difference in expression level between the HFD and HFD + NMN-treated groups. Data were filtered for log_2_FC above 0.3 or below −0.3 and *p* value (Limma) < 0.05. The log_2_FC ranged from −1.402 on the under-expression side and 1.618 on the overexpression side. Among these proteins, 19 proteins were upregulated and 17 proteins were downregulated. The top five upregulated proteins were Acnat2 (log_2_FC = 1.62, *p* = 0.024), Cyp2b10 (log_2_FC = 1.24, *p* = 0.003), Cyp2b9 (log_2_FC = 1.03, *p* = 0.023), Atxn7l3b (log_2_FC = 0.73, *p* = 0.016), and Creld1 (log_2_FC = 0.57, *p* = 0.002). The most significantly downregulated protein was Ces3b (carboxylesterase 3B) with a log_2_FC of −0.54 and *p* < 0.005. Other proteins with significantly affected expression were Atp2a1 (log_2_FC = −1.40, *p* = 0.005), Apex1 (log_2_FC = −0.30, *p* = 0.005), Clic4 (log_2_FC = −0.31, *p* = 0.0002), Cml5 (log_2_FC = −0.54, *p* = 0.029), Ctsf (log_2_FC = −0.34, *p* = 0.023), and Ctsz (log_2_FC = −0.31, *p* = 0.023), which were downregulated, and Aldh1a1 (log_2_FC = 0.41, *p* = 0.0001) and Akr1d1 (log_2_FC = 0.44, *p* = 0.001), which were upregulated.

**Figure 3:**
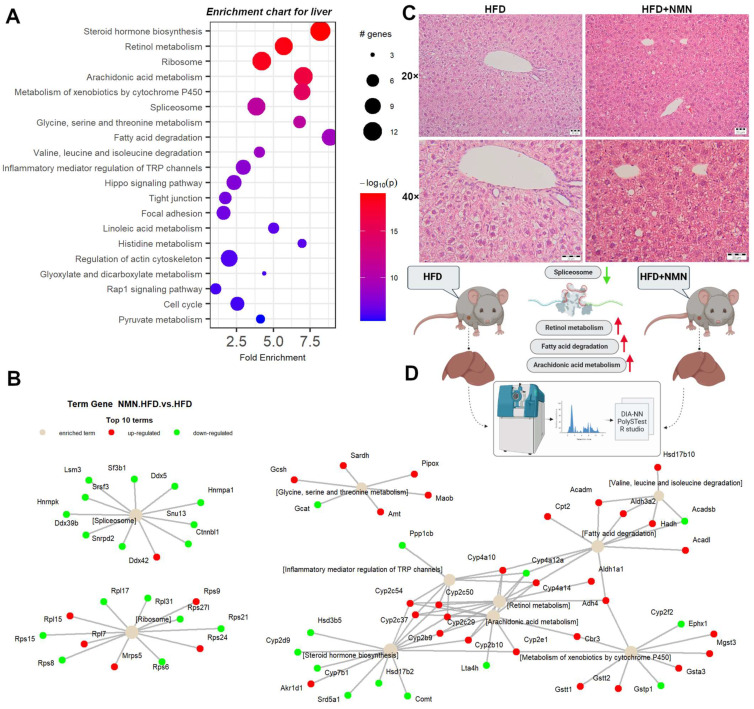
Integrated proteomics data analysis of NMN-treated HFD mouse liver. (**A**) Enrichment chart for top 20 KEGG Pathways sorted by lowest *p* value. (**B**) Term–gene graph for top 10 terms. (**C**) Representative images of hematoxylin and eosin staining for mouse liver tissue. Scale bars, 20 μm. (**D**) Experiment summary (BioRender).

Enrichment chart (Figure 3A) and term–gene graph (Figure 3B) analysis generated in PathfindR from mouse liver proteomics data revealed several biological pathways that were significantly affected. The top affected pathways were steroid hormone biosynthesis, retinol metabolism, ribosome, arachidonic acid metabolism, metabolism of xenobiotics by cytochrome P450, spliceosome, fatty acid degradation, and inflammatory mediator regulation of TRP channels. The steroid hormone biosynthesis pathway (Figure S1) was significantly altered with upregulated proteins, including Akr1d1, Cyp2b10, Cyp2b9, Cyp2c29, Cyp2c37, Cyp2c50, Cyp2c54, and Cyp2e1. Similarly, the retinol metabolism pathway was affected by upregulated proteins such as Adh4, Aldh1a1, Cyp2b10, Cyp2b9, Cyp2c29, Cyp2c37, Cyp2c50, Cyp2c54, and Cyp4a10. On the other hand, the ribosome pathway (Figure S2) showed upregulated proteins such as Mrps5, Rps9, Rps24, Rpl7, and Rpl15, while proteins Rps6, Rps8, Rps15, Rps21, Rps27l, Rpl17, and Rpl31 were downregulated. The arachidonic acid metabolism pathway (Figure S3) showed upregulated proteins such as Cbr3, Cyp2e1, Cyp4a10, Cyp4a14, Cyp2b10, Cyp2b9, Cyp2c29, Cyp2c37, and Cyp2c54 and downregulated proteins such as Lta4h and Cyp4a12a. Similarly, the metabolism of xenobiotics by the cytochrome P450 pathway was affected with upregulated proteins such as Gsta3, Gstt1, Gstt2, Mgst3, Ephx1, Cyp2e1, Cbr3, and Adh4, while proteins Gstp1 and Cyp2f2 were downregulated.

Other upregulated pathways were inflammatory mediator regulation of TRP channels, the Hippo signaling pathway, glycine, serine and threonine metabolism, fatty acid degradation, valine, leucine and isoleucine degradation, linoleic acid metabolism, histidine metabolism, regulation of actin cytoskeleton, glyoxylate and dicarboxylate metabolism, and pyruvate metabolism. Downregulated proteins were observed in tight junction, focal adhesion, the Rap1 signaling pathway, and cell cycle pathways.

### 2.3. Thermogenesis Pathway Is Upregulated by NMN in Muscle Tissue

Skeletal muscle is a highly specialized tissue that is essential for movement and controls the body’s whole glucose metabolism. It is responsible for 75–80% of glucose uptake during hyperinsulinemia, followed by adipose tissue and the liver. The complete list of identified proteins including log_2_ fold change (log_2_FC), *p* value, and expression values for each individual and technical replicate is provided in the Supplemental Information.

**Figure 4:**
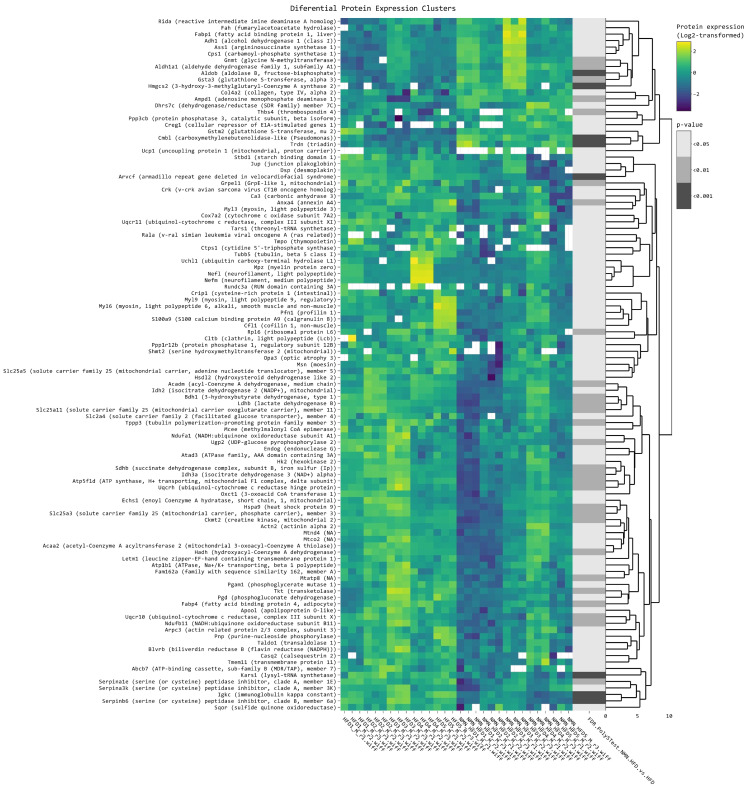
Clustered heatmap of the differentially expressed proteins in mouse skeletal muscle tissue. Clustered heatmap of the 119 differentially expressed proteins in mouse muscle tissue, filtered with log_2_FC threshold set to exclude the interval −0.3, 0.3, allowing *p* < 0.05. Yellow color represents upregulation, while blue represents downregulation in HFD + NMN-treated group compared to HFD group. From left to right, expression values (log_2_ transformed) for replicates (5 biological × 3 technical) are shown for the HFD group and for the HFD + NMN-treated group, followed by significance values of the comparison to HFD group.

A clustered heatmap of the 119 differentially expressed proteins in all samples (five biological × three technical replicates in each condition/diet) (Figure 4) shows proteins grouped by several similar expression patterns. The proteins in the first small cluster are grouped in three subclusters. Among these, Gnmt, Aldob, and Hmgcs2 had the highest log_2_FC values of 1.39, 1.21, and 1.06, respectively. The second subcluster has six differentially expressed proteins, with Thbs4 having the highest log_2_FC of 0.76. The third subcluster has four differentially expressed genes, with Ucp1 having the highest log_2_FC of 1.64 among all the identified proteins (Table S2).

Analysis of the second and the larger cluster revealed significant changes in the expression of 38 proteins, with log_2_FC ranging from −1.8576 to −0.3068 and *p* values ranging from 0.00098 to 0.04585. The most significantly downregulated protein expression was that of Arvcf (log_2_FC = −1.86), followed by S100a9 (log_2_FC = −1.64), Mpz (log_2_FC = −1.58), and Nefl (log_2_FC = −1.40). We also observed downregulation of several other proteins involved in muscle function, including Myl3 (log_2_FC = −1.44), Myl9 (log_2_FC = −0.53), Myl6 (log_2_FC = −0.40), and Tubb5 (log_2_FC = −0.42). Moreover, we observed downregulation of genes involved in mitochondrial function, including Cox7a2 (log_2_FC = −0.32), Uqcr11 (log_2_FC = −0.52), and Slc25a5 (log_2_FC = −0.31) (Table S2).

**Figure 5:**
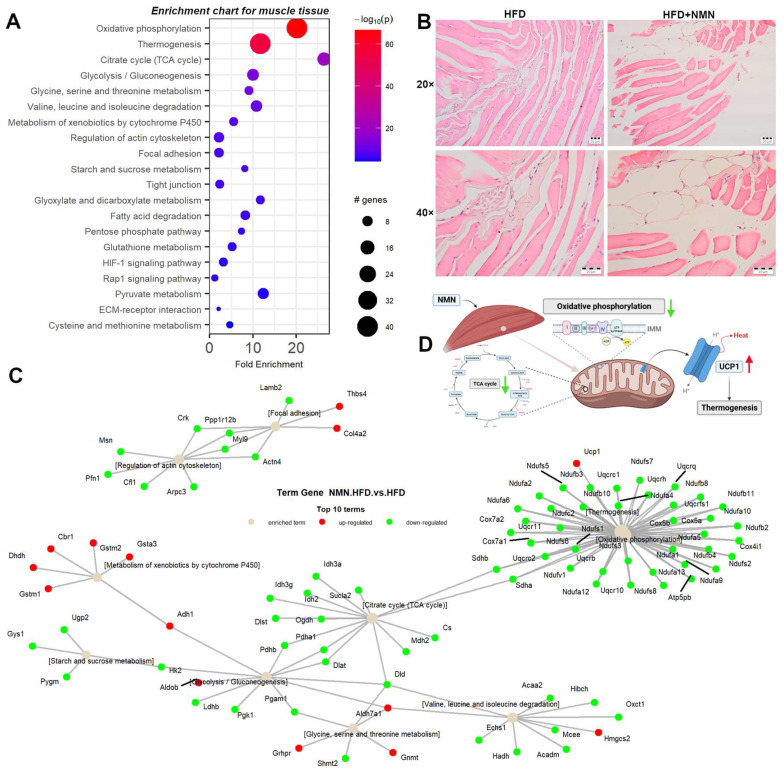
Integrated proteomics data analysis of mouse muscle tissue. (**A**) Enrichment chart for top 20 KEGG Pathways sorted by lowest *p* value in mouse muscle tissue. (**B**) Representative images of hematoxylin and eosin staining and semiquantitative analysis from mouse muscle tissue. Scale bars, 20 μm. (**C**) Term–gene graph for top 10 terms in mouse liver. (**D**) Experiment summary (BioRender).

Enrichment pathway analysis (Figure 5A,C) shows downregulated proteins involved in mitochondrial functions, including oxidative phosphorylation and the TCA cycle. We observed downregulation of proteins involved in glycolysis/gluconeogenesis, glycine/serine/threonine metabolism, valine/leucine/isoleucine degradation, metabolism of xenobiotics by cytochrome P450, regulation of actin cytoskeleton, focal adhesion, fatty acid degradation, and cysteine and methionine metabolism. Additionally, some pathways showed both upregulated and downregulated proteins, such as glycolysis/gluconeogenesis (Figure S7), glycine/serine/threonine metabolism (Figure S8), and pyruvate metabolism (Figure S9). Moreover, we identified specific proteins that were significantly upregulated or downregulated in certain pathways, such as Aldob, Adh1, and Aldh7a1 in glycine, serine, and threonine metabolism and the pentose phosphate pathway, Ucp1 in thermogenesis, and Aldh7a1 and Hmgcs2 in valine, leucine, and isoleucine degradation (Figure S10). Notably, metabolism of xenobiotics by cytochrome P450 was significantly upregulated in the muscle tissue of NMN-treated mice (Figure 5C).

### 2.4. NMN Stimulates Adipose Cell Proliferation by Upregulating mTOR Pathway

**Figure A1:**
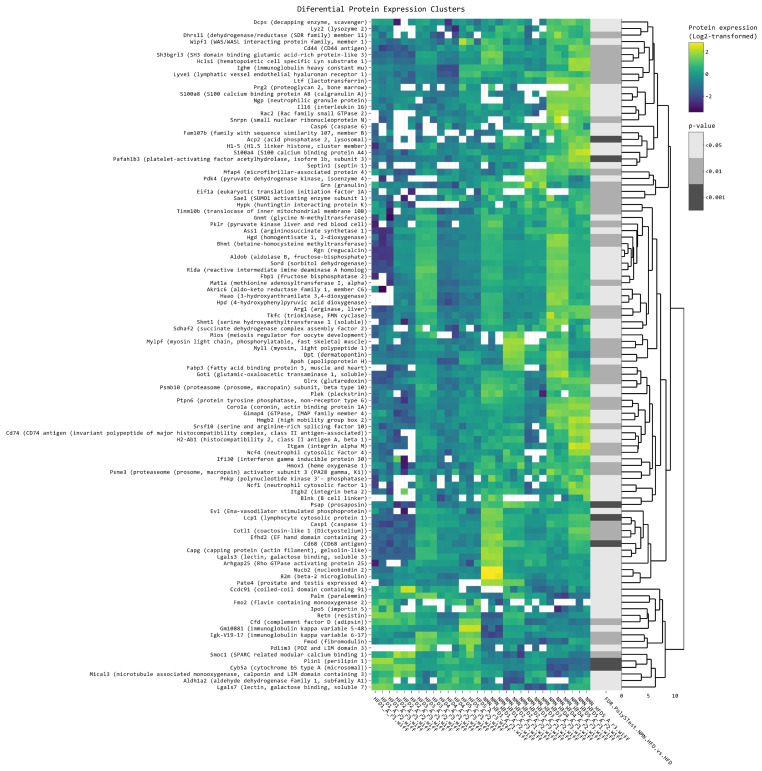
Clustered heatmap of the differentially expressed proteins in mouse adipose tissue. Clustered heatmap of the 119 differentially expressed proteins, filtered with log_2_FC threshold set to exclude the interval −0.5, 0.5 and 0.05 *p* threshold. Yellow color represents upregulation, while blue represents downregulation in NMN-treated group versus HFD group. From left to right, expression values (log_2_ transformed) for replicates (5 biological × 3 technical) are shown for the HFD group and for the HFD + NMN-treated group, followed by significance values of the comparison to HFD group.

The heatmap analysis of mouse adipose tissue (Figure A1) revealed significant changes in a cluster formed by 104 proteins (Table S3). Among the significantly upregulated proteins, the most highly induced were Aoc1 (amine oxidase, copper-containing 1) with a log_2_FC of 4.85 and *p* < 0.03, followed by Epx (eosinophil peroxidase) with a log_2_FC of 2.16 and *p* < 0.01, and Aldob (aldolase B, fructose-bisphosphate) with a log_2_FC of 1.40 and *p* < 0.01. The significantly downregulated proteins included Fut11 (fucosyltransferase 11) with a log_2_FC of −1.04 and *p* < 0.002, followed by H1-5 (H1.5 linker histone, cluster member) with a log_2_FC of −0.50 and *p* < 0.04, and Gnmt (glycine N-methyltransferase) with a log_2_FC of 1.15 and *p* < 0.01.

**Figure 6:**
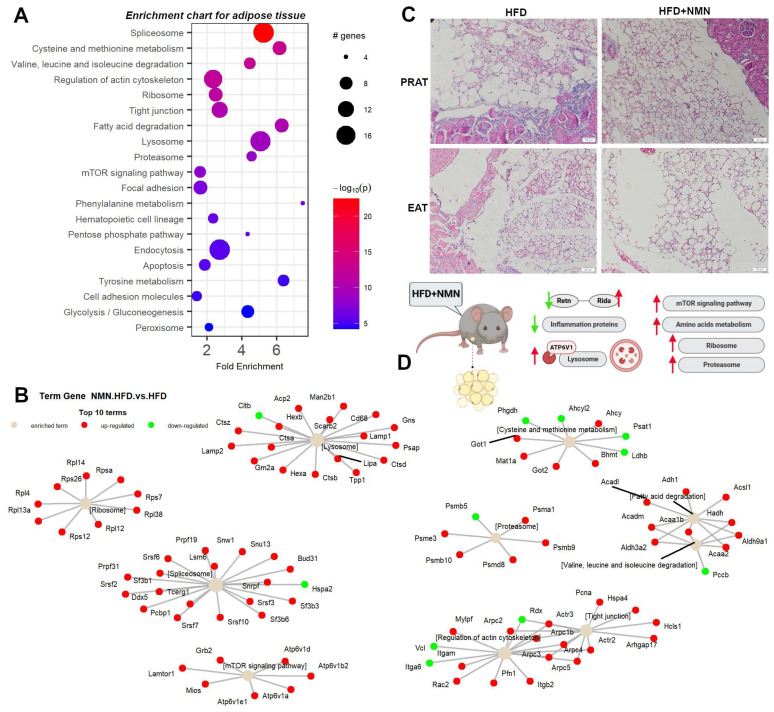
Integrated proteomics data analysis of NMN-treated HFD mouse adipose tissue. (**A**) Enrichment chart for top 20 KEGG Pathways sorted by lowest *p* value in mouse adipose tissue. (**B**) Term–gene graph for top 10 terms. (**C**) Representative images of hematoxylin and eosin staining for perirenal adipose tissue (PRAT) and epicardial adipose tissue (EAT). Scale bars, 50 μm. (**D**) Experiment summary (BioRender).

In the second and the smallest cluster, sixteen proteins were found to have significantly altered expression levels (*p* < 0.05). Of these, 15 proteins showed a decrease in expression in response to NMN treatment, with log_2_FC ranging from −0.973 to −0.503. One of the proteins significantly repressed was Resistin, with a log_2_FC of −0.78 and *p* < 0.045. Also, other proteins that showed significant changes in expression levels were Cfd (complement factor D), Gm10881 (immunoglobulin kappa variable 5–48), Igk-V19-17 (immunoglobulin kappa variable 6–17), and Cyb5a (cytochrome b5 type A (microsomal)). These proteins are involved in various physiological processes such as immune response, lipid metabolism, and oxidative stress response (Figure 6A). The upregulation of Aoc1, Epx, and Aldob suggests increased oxidative stress and lipid metabolism, whereas the downregulation of Fut11, H1-5, Gnmt, Cltb, and Hspa2 indicates altered cellular processes such as immune response and DNA packing (Figure 6B). Moreover, the following enrichment chart (Figure 6A) and term–gene graph (Figure 6B) reveal significant changes in the expression level of proteins involved in various biological processes and pathways: the spliceosome pathway (Figure S11) showed upregulation of Snrpf, Ddx5, Tcerg1, Sf3b1, Sf3b3, Sf3b6, Lsm6, Prpf31, Snu13, Prpf19, Snw1, Bud31, Pcbp1, Srsf2, Srsf3, Srsf6, Srsf7, and Srsf10, while Hspa2 was downregulated.

In cysteine and methionine metabolism pathways, Bhmt, Mat1a, Ahcy, Got1, and Got2 were upregulated while Ahcyl2, Ldhb, Phgdh, and Psat1 were downregulated. Valine, leucine, and isoleucine degradation was altered with Acadm, Hadh, Acaa1b, Acaa2, Aldh3a2, and Aldh9a1 being upregulated, while Pccb was downregulated. At the level of the actin cytoskeleton (Figure S12), upregulation of Itgam, Itgb2, Rac2, Mylpf, Actr2, Actr3, Arpc1b, Arpc2, Arpc3, Arpc4, Arpc5, and Pfn1 was noticed, while Itga6, Rdx, and Vcl were downregulated (Figure 6B). The ribosome pathway showed upregulation as component proteins like Rps7, Rps12, Rps26, Rpsa, Rpl4, Rpl12, Rpl13a, Rpl14, and Rpl38 were upregulated. At the level of tight junctions, overexpression of Arhgap17, Pcna, Hspa4, Hcls1, Actr2, Actr3, Arpc1b, Arpc2, Arpc3, Arpc4, and Arpc5 was observed, while Rdx was downregulated. Fatty acid degradation showed upregulation by overexpression of Acaa1b, Acaa2, Hadh, Acadm, Acadl, Acsl1, Adh1, Aldh3a2, and Aldh9a1. Most of the lysosome pathway components were upregulated (Ctsa, Ctsb, Ctsd, Ctsz, Tpp1, Hexa, Hexb, Man2b1, Gns, Lipa, Acp2, Psap, Gm2a, Lamp1, Lamp2, Cd68, and Scarb2) while Cltb was downregulated. The proteasome pathway (Figure S13) showed upregulation of Psmd8, Psme3, Psma1, Psmb9, and Psmb10, while Psmb5 was downregulated. The mTOR signaling pathway was upregulated in adipose tissue by overexpression of Atp6v1a, Atp6v1b2, Atp6v1d, Atp6v1e1, Lamtor1, Mios, and Grb2. The focal adhesion pathway showed upregulation of Lama2, Mylpf, Rac2, and Grb2, while Lama4, Itga6, Ilk, Vcl, and Cav1 were downregulated.

### 2.5. Downregulated OXPHOS Proteins and Upregulated Ketone Bodies Production Were Shown in Brains of NMN-Treated HFD Mice 

**Figure 7:**
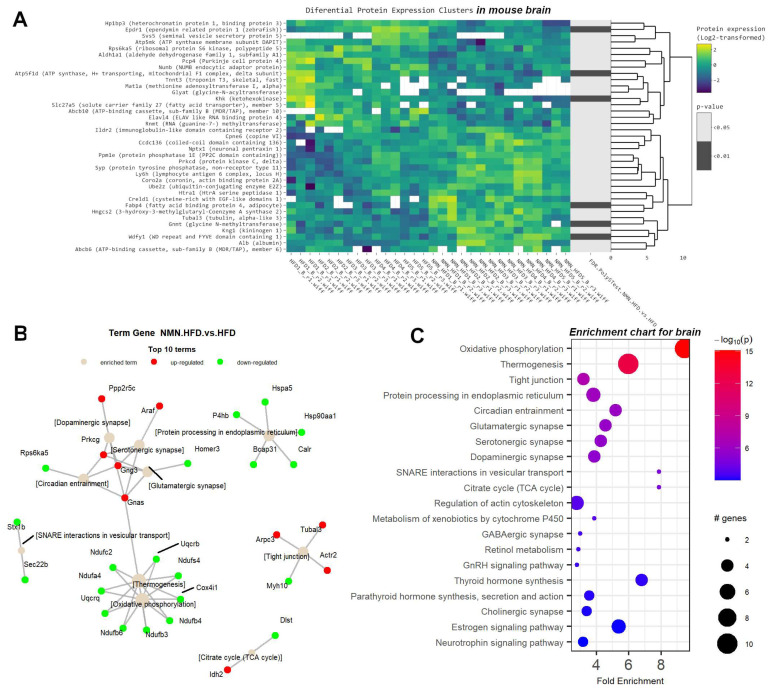
Significant changes induced by NMN treatment in the HFD mouse brain proteome. (**A**) Clustered heatmap of the 45 differentially expressed proteins, filtered with log_2_FC threshold set to exclude the interval −0.2, 0.2; 0.05 *p* value threshold. Yellow color represents upregulation, while blue represents downregulation in HFD + NMN-treated group compared to HFD group. From left to right, expression values (log_2_ transformed) for replicates (5 biological × 3 technical) are shown for the HFD group and for the HFD + NMN-treated group, followed by significance values of the comparison to HFD group. (**B**) Term–gene graph for top 10 terms. (**C**) Enrichment chart for top 20 KEGG pathways sorted by lowest *p* value.

We performed heatmap proteomics analysis (Figure 7A) of mouse brain samples to compare the protein expression between the HFD group and the HFD + NMN-treated group. We identified a total of 45 differentially expressed proteins (Table S4) with a *p* value < 0.05 (Limma test) Among these, 23 proteins were downregulated and 14 proteins were upregulated in the HFD + NMN group compared to the HFD group. The most significantly downregulated protein was Svs5 (seminal vesicle secretory protein 5) with a log_2_FC of −2.02 (*p* = 0.011). Other significantly downregulated proteins included Tnnt3 (troponin T3, skeletal, fast), Glyat (glycine-N-acyltransferase), and Slc27a5 (solute carrier family 27 (fatty acid transporter), member 5) with log_2_FC values of −1.23, −1.18, and −0.51, respectively. The most significantly upregulated protein was Creld1 (cysteine-rich with EGF-like domains 1) with a log_2_FC of 0.80 (*p* = 0.04). Other significantly upregulated proteins included Gnmt (glycine N-methyltransferase), Alb (albumin), and Abcb6 (ATP-binding cassette, sub-family B (MDR/TAP), member 6) with log_2_FC values of 0.82, 0.33, and 0.38, respectively.

The term–gene graph (Figure 7B) and enrichment chart (Figure 7C) above suggest that NMN treatment induces significant changes in the mouse brain proteome in the expression of proteins involved in various biological processes including fatty acid transport, energy metabolism, and signal transduction. These findings provide insights into the molecular mechanisms underlying the beneficial effects of HFD + NMN treatment on brain function. One of the most affected pathways was oxidative phosphorylation (Figure S16), with Ndufs4, Ndufa4, Ndufb3, Ndufb4, Ndufb6, Ndufc2, Uqcrb, Uqcrq, and Cox4i1 identified as downregulated proteins. Thermogenesis was mostly downregulated in the brain, as it shares many proteins with the OXPHOS pathway, excluding Gnas which was identified as upregulated. Tight junctions (Figure S17) were also affected, with Actr2, Arpc3, and Tubal3 identified as upregulated and Myh10 as downregulated. Protein processing in endoplasmic reticulum (Figure S18) members like Hspa5, Calr, P4hb, Bcap31, and Hsp90aa1 was upregulated. The circadian pathway was mostly upregulated by the member proteins Gng3, Gnas, and Prkcg, while Rps6ka5 was downregulated. Glutamatergic synapse and dopaminergic synapse pathways were also affected, with Prkcg, Gnas, and Gng3 identified as upregulated proteins in both pathways. SNARE interactions in the vesicular transport pathway were also modified, with Stx1b and Sec22b downregulated. Finally, the citrate cycle (TCA cycle) had Idh2 identified as upregulated and Dlst as downregulated.

### 2.6. NMN Treatment Decreased Mitochondrial Function with Increased Membrane Potential and Higher ROS Production in Muscle Cells while in Hepatic Cells, Mitochondrial Mass Was Higher and Mitochondrial Membrane Potential Was Reduced

**Figure A2:**
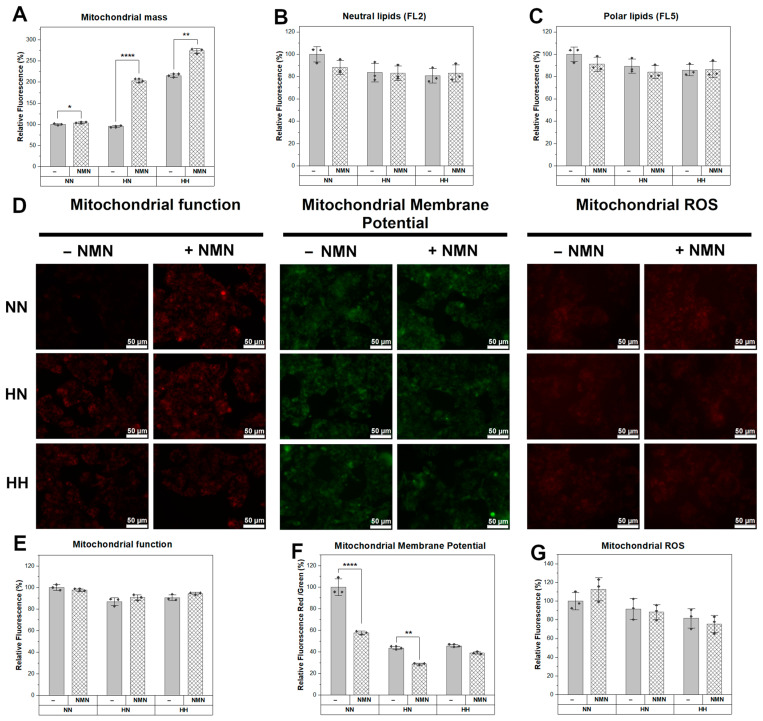
NMN effects on HepG2 cells exposed to hyperglycemic conditions: neutral lipids, polar lipids, mitochondrial mass, metabolic activity, membrane potential, and reactive oxygen species (ROS). (**A**) Mitochondrial mass with 10-nonyl-acridine orange (NAO). (**B**) Neutral lipids with Nile Red (FL2). (**C**) Polar lipids with Nile Red (FL5). (**D**) Fluorescence microscopy. Scale bars, 50 μm. (**E**) Mitochondrial function with MitoView Red. (**F**) Mitochondrial membrane potential with JC-1. (**G**) Mitochondrial ROS with DHR-123 in C2C12 myotubes during 100 μM NMN treatment in normoglycemic conditions (NN), hyperglycemic conditions followed by culture media switch to normoglycemic conditions during NMN treatment (HN), hyperglycemic conditions before and during treatment (HH), versus untreated condition (−). The flow cytometry data are illustrated as average values of the groups (*n* = 3 × 15,000 events) ± standard deviation of the mean (STDEV) and statistical significance between NMN-treated and untreated conditions. * *p* < 0.05; ** *p* < 0.01; **** *p* < 0.0001.

**Figure A3:**
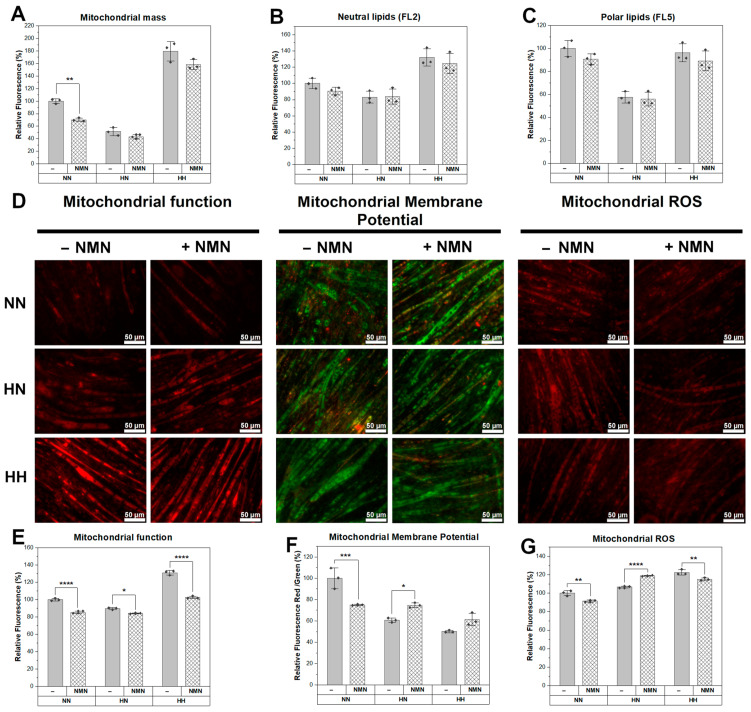
NMN effects on C2C12-derived myotubes exposed to hyperglycemic conditions: neutral lipids, polar lipids, mitochondrial mass, metabolic activity, membrane potential, and reactive oxygen species (ROS). (**A**) Mitochondrial mass with 10-nonyl-acridine orange (NAO). (**B**) Neutral lipids with Nile Red (FL2). (**C**) Polar lipids with Nile Red (FL5). (**D**) Fluorescence microscopy. Scale bars, 50 μm. (**E**) Mitochondrial function with MitoView Red. (**F**) Mitochondrial membrane potential with JC-1. (**G**) Mitochondrial ROS with DHR-123 in C2C12 myotubes during 100 μM NMN treatment in normoglycemic conditions (NN), hyperglycemic conditions followed by culture media switch to normoglycemic conditions during NMN treatment (HN), hyperglycemic conditions before and during treatment (HH), versus untreated condition (−). The flow cytometry data are illustrated as average values of the groups (*n* = 3 × 15,000 events) ± standard deviation of the mean (STDEV) and statistical significance between NMN-treated and untreated conditions. * *p* < 0.05; ** *p* < 0.01; *** *p* < 0.001; **** *p* < 0.0001.

The results of the flow cytometry analysis for HepG2 cells and C2C12 myotubes in six experimental conditions are presented in Figure A2 and Figure A3. The conditions tested were normoglycemic (NN), hyperglycemic followed by culture media switch to normoglycemic during NMN treatment (HN), and hyperglycemic before and during treatment (HH), treated with 100 μM NMN versus untreated, and the parameters analyzed were mitochondrial mass, mitochondrial function, mitochondrial membrane potential, mitochondrial ROS, intracellular neutral lipids, and intracellular polar lipids.

In accordance with the in vivo experiment, we investigated the influence of NMN treatment in HepG2 cells (as an in vitro liver model for T2DM) by flow cytometry. NMN treatment led to a significant increase in mitochondrial mass (Figure A2A) by 3.56% in NN conditions (*p* < 0.05), 113% in HN conditions (*p* < 0.0001), and 27.38% in HH conditions (*p* < 0.01). There was no significant change in neutral lipids (Figure A2B), polar lipids (Figure A2C), mitochondrial function (Figure A2E), or mitochondrial ROS (Figure A2G). Mitochondrial membrane potential (Figure A2F) was significantly decreased by 42.33% (*p* < 0.0001) and 33.91% (*p* < 0.01) in NN and HN conditions, respectively, after NMN treatment. No significant changes in mitochondrial membrane potential were observed in HH conditions.

The effects of NMN treatment on mitochondrial parameters were evaluated in differentiated C2C12 myotubes. The statistical significance was calculated for each parameter by comparing the treated and untreated conditions. Regarding mitochondrial mass (Figure A3A) in NN conditions, NMN treatment resulted in a significant decrease in mitochondrial mass by 29.73% (*p* < 0.01). However, NMN treatment induced no significant changes in mitochondrial mass, neutral lipids (Figure A3B), or polar lipids (Figure A3C). NMN treatment led to a significant decrease in mitochondrial function (Figure A3E) by 14.77% (*p* < 0.0001) in NN conditions, 6.34% (*p* < 0.05) in HN conditions, and 21.76% (*p* < 0.0001) in HH conditions. Mitochondrial membrane potential (Figure A3F) was significantly decreased by 24.95% (*p* < 0.001) in NN conditions but significantly increased by 23.27% (*p* < 0.05) in HN conditions after NMN treatment. However, no significant changes in mitochondrial membrane potential were induced by NMN treatment in HH conditions. Mitochondrial ROS (Figure A3G) were significantly decreased by 8.43% and 6.11% (*p* < 0.01) in NN and HH conditions, respectively, and significantly increased by 11.39% (*p* < 0.0001) in HN conditions after NMN treatment.

### 2.7. NMN Downregulates Spliceosome Proteins While Upregulating Ribosome Proteins in Hepatocytes

**Figure A4:**
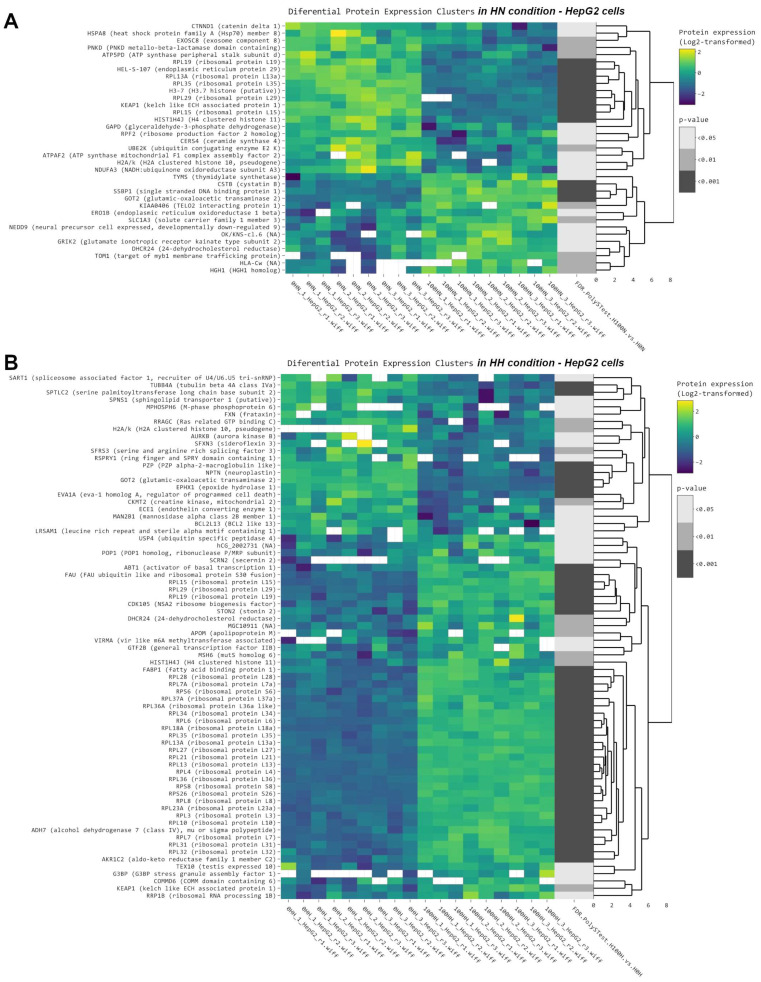
Clustered heatmap of the differentially expressed proteins in HepG2 cells. (**A**) Clustered heatmap of the 37 common differentially expressed proteins in HN conditions, filtered with log_2_FC threshold set to exclude the interval −0.3, 0.3, 0.05 *p* threshold. From left to right, expression values (log_2_ transformed) for replicates (3 biological × 3 technical) are shown for the 0HN group and for the 100HN (treated) group, followed by significance values of the comparison to 0HN group. (**B**) Clustered heatmap of the 85 differently expressed proteins in HH conditions, filtered with log_2_FC threshold set to exclude the interval −0.3, 0.3 and 0.05 *p* threshold. From left to right, expression values (log_2_ transformed) for replicates (3 biological × 3 technical) are shown for the 0HH (untreated) group and for the 100HH (treated) group, followed by significance values of the comparison to 0HH group.

The heatmap analysis of NMN-treated HepG2 cells under HN conditions (Figure A4A) revealed downregulation of ATP5PD, ATPAF2, CERS4, CTNND1, EXOSC8, GAPD, H2A/k, H3-7, HEL-S-107, HIST1H4J, KEAP1, NDUFA3, RPF2, RPL13A, RPL15, RPL19, RPL29, and RPL35 proteins while DHCR24, GOT2, GRIK2, HGH1, HLA-Cw, KIAA0406, NEDD9, OK/KNS-cl.6, PNKD, SLC1A3, SSBP1, TOM1, TYMS, and UBE2K proteins were significantly upregulated.

On the other hand, heatmap analysis of NMN-treated HepG2 cells under HH conditions (Figure A4B) revealed significant changes in the expression of 85 proteins (Table S5) with a *p* value < 0.05 and a log_2_FC below −0.3 or above 0.3. Of these, 56 proteins were upregulated, while 29 were downregulated in the HH condition treated with NMN (H100H) compared to the untreated control (H0H condition). Among the upregulated proteins were AP2B1, FABP1, FAU, and GALT, while downregulated proteins included ATE1, AURKB, EPHX1, and NGEF. We observed changes in proteins involved in diverse biological processes, including protein synthesis (CDK105), signal transduction (G3BP), and carbohydrate metabolism (GOT2).

**Figure 8:**
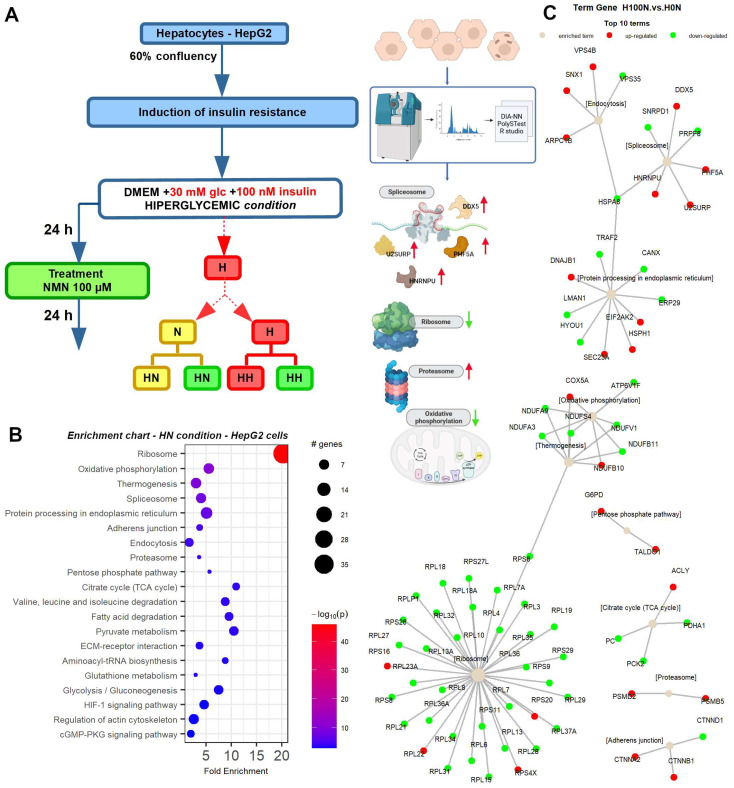
Integrated proteomics data analysis of HepG2 cell line under HN conditions. (**A**) Experiment summary (OpenOffice, BioRender). (**B**) Enrichment chart for top 20 KEGG Pathways in HN conditions for HepG2 cells sorted by lowest *p* value. (**C**) Term–gene graph for top 10 terms in HN conditions for HepG2 cells.

**Figure A5:**
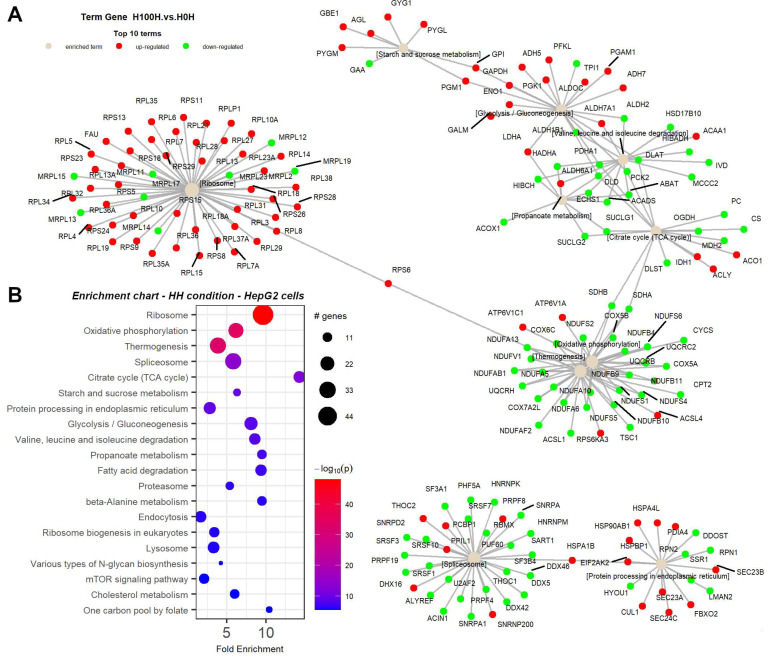
Integrated proteomics data analysis of HepG2 cell line under HH conditions. (**A**) Term–gene graph for top 10 terms in HH conditions for HepG2 cells. (**B**) Enrichment chart for top 20 KEGG pathways in HH conditions for HepG2 cells sorted by lowest *p* value.

Pathway enrichment analysis (Figure 8B and Figure A5B) showed that the ribosome pathway (Figure S19) was significantly influenced in both HN and HH conditions. In HN conditions (Figure 8C), RPS4X, RPS16, RPS20, and RPL22 were upregulated, while 29 ribosomal proteins were downregulated. In contrast, in HH conditions (Figure A5A), RPS5, RPS6, RPS8, RPS9, RPS11, RPS13, RPS15, RPS18, RPS23, RPS24, RPS26, RPS28, RPS29, FAU, MRPL2, and 28 ribosomal proteins were upregulated, while MRPL12, MRPL11, MRPL13, MRPL14, MRPL15, MRPL17, MRPL19, and MRPL23 were downregulated.

The oxidative phosphorylation pathway (Figure S20) was also significantly influenced in both conditions, with NDUFB10 and COX5A upregulated in HN conditions. Moreover, ATP6V1A and ATP6V1C1 were upregulated in HH conditions. Interestingly, the OXPHOS pathway was affected in both HN and HH conditions, but the set of up- and downregulated proteins was different. In HN conditions, NDUFB10 and COX5A were upregulated, while RPS6, NDUFS4, NDUFV1, NDUFA3, NDUFA9, and NDUFB11 were downregulated. In HH conditions, RPS6KA3 and ACSL4 were upregulated, while TSC1, ACSL1, and many other proteins involved in oxidative phosphorylation were downregulated.

The spliceosome pathway was significantly influenced by NMN treatment only in HN conditions, with DDX5, PHF5A, U2SURP, and HNRNPU upregulated and SNRPD1, PRPF8, and HSPA8 downregulated. In HH conditions, several spliceosome proteins were downregulated, but the pathway was not significantly influenced (Figure A5A,B and Figure S21). Finally, the citrate cycle and valine, leucine, and isoleucine degradation pathways were significantly influenced only in HH conditions, with different sets of up- and downregulated proteins in each pathway.

### 2.8. NMN Downregulates Proteasome and Upregulates DNA Replication and Cell Cycle Pathways in Muscle Cells

**Figure A6:**
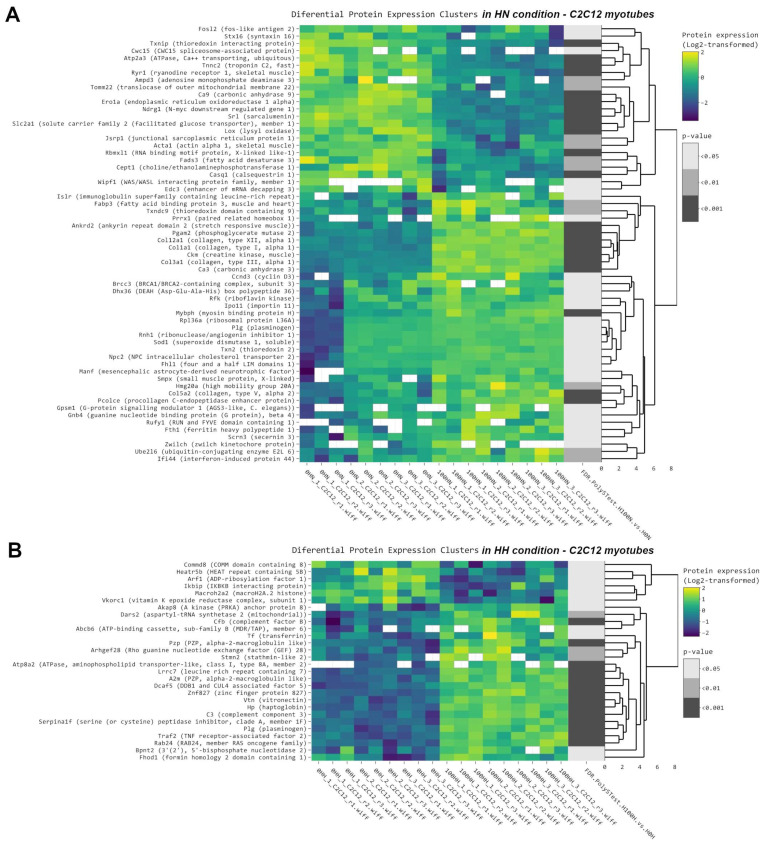
Clustered heatmap of the differentially expressed proteins in myotubes. (**A**) Clustered heatmap of the 63 differentially expressed proteins in HN conditions, filtered with log_2_FC threshold set to exclude the interval −0.3, 0.3 and 0.05 *p* threshold. From left to right, expression values (log_2_ transformed) for replicates (3 biological × 3 technical) are shown for the 0HN (untreated) group and for the 100HN (treated) group, followed by significance values of the comparison to 0HN (untreated) group. (**B**) Clustered heatmap of the 30 common differentially expressed proteins in HH conditions, filtered with log_2_FC threshold set to exclude the interval −0.3, 0.3 and 0.05 *p* threshold. From left to right, expression values (log_2_ transformed) for replicates (3 biological × 3 technical) are shown for the 0HH group and for the 100HH (treated) group, followed by significance values of the comparison to 0HH (untreated) group.

Heatmap analysis of C2C12 myotubes in HN conditions (Figure A6A) revealed differential expression of several proteins upon treatment with 100 μM NMN (H100N) compared to the untreated condition (H0N). Out of the 63 proteins (Table S6), the most significantly upregulated protein was Islr with a log_2_FC of 0.79 and an *p* value of 0.026. On the other hand, the most significantly downregulated protein was Hgsnat with a log_2_FC of −1.18 and a *p* value of 0.022. Among the upregulated proteins, Ankrd2 had the highest log_2_FC (0.35) and the lowest *p* value (1.2 × 10^−6^). Among the downregulated proteins, Ash2l had the lowest log_2_FC (−0.89), while Casq1 had the lowest *p* value (0.0001).

Moreover, several proteins related to muscle function showed significantly different expression levels in myotubes upon treatment with NMN (H100N) compared to H0N. Creatine kinase (Ckm) and myosin-binding protein H (Mybph) were upregulated with log_2_FC values of 0.36 and 0.33, respectively. In contrast, actin alpha 1, skeletal muscle (Acta1) was downregulated with a log_2_FC of −0.44. Other extracellular matrix proteins such as collagen, type I, alpha 1 (Col1a1) and collagen, type III, alpha 1 (Col3a1) were upregulated with log_2_FC values of 0.34 and 0.43, respectively.

To investigate the effect of NMN treatment on C2C12 myotubes in hyperglycemic conditions (HH), we conducted proteomics data analysis by heatmap and pathway enrichment. Among the 30 proteins from H100H versus H0H comparisons in the heatmap (Figure A6B), 23 were upregulated and 7 were downregulated due to NMN treatment. The protein with the largest upregulation was Abcb6 (ATP-binding cassette, sub-family B (MDR/TAP), member 6) with a log_2_FC of 0.624 and *p* < 0.015. Other significantly upregulated proteins included Man1a2 (mannosidase, alpha, class 1A, member 2), A2m (PZP, alpha-2-macroglobulin like), and Vtn (vitronectin). On the other hand, the most repressed protein was Pdp1 (pyruvate dehydrogenase phosphatase catalytic subunit 1) with a log_2_FC of −0.953 and *p* < 0.02. Other significantly downregulated proteins included Macroh2a2 (macroH2A.2 histone) and Heatr5b (HEAT repeat containing 5B).

**Figure A7:**
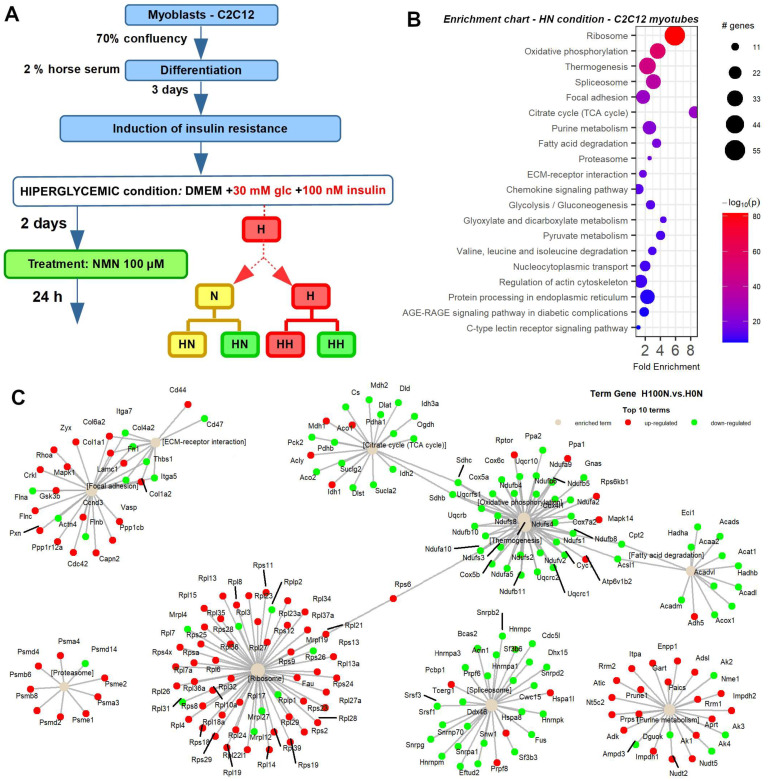
Integrated proteomics data analysis of myotubes under HN conditions. (**A**) Experiment summary (OpenOffice) in myotubes. (**B**) Enrichment chart for top 20 KEGG pathways in HN conditions for myotubes cells sorted by lowest *p* value. (**C**) Term–gene graph for top 10 terms in HN conditions for C2C12-derived myotubes.

**Figure A8:**
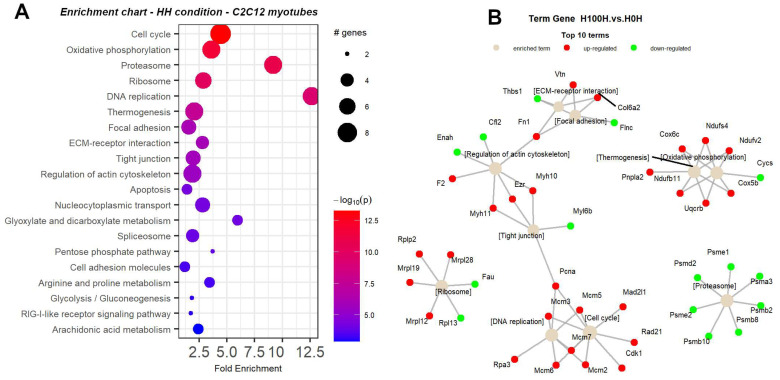
Integrated proteomics data analysis of myotubes under HH conditions. (**A**) Enrichment chart for top 20 KEGG pathways in HH conditions for C2C12-derived myotubes sorted by lowest *p* value. (**B**) Term–gene graph for top 10 terms in HH conditions for C2C12 myotubes.

To better understand the biological significance of the differentially expressed proteins identified, we performed pathway enrichment analysis using PathfindR. The results of the enrichment chart (Figure A7B and Figure A8A) show several functional pathways, including amino acid metabolism, cytoskeleton organization, protein processing, and energy metabolism.

A term–gene graph revealed significant changes in protein expression in various pathways: ribosome-related proteins (Figure S23) were upregulated in HN conditions (Figure A7C), including Rps2, Rps4x, Rps6, Rps8, Rps9, Rps11, Rps12, Rps13, Rps18, Rps19, Rps23, Rps24, Rps25, Rps26, Rps29, Fau, Rpsa, Rpl3, Rpl4, Rpl6, Rpl7, Rpl7a, Rpl8, Rpl10a, Rpl13, Rpl13a, Rpl14, Rpl15, Rpl17, Rpl18a, Rpl19, Rpl21, Rpl22l1, Rpl23, Rpl23a, Rpl24, Rpl26, Rpl27, Rpl27a, Rpl28, Rpl29, Rpl32, Rpl34, Rpl35, Rpl36, Rpl37a, and Rpl39. In contrast, several ribosome-related proteins were downregulated, including Rps28, Mrpl4, Mrpl12, Mrpl19, Mrpl27, Rpl31, and Rplp1 in HN conditions. The cell cycle pathway was upregulated by NMN in HH conditions, including Cdk1, Rad21, Mad2l1, Pcna, Mcm2, Mcm3, Mcm5, Mcm6, and Mcm7 (Figure A8B and Figure S24).

Also, the oxidative phosphorylation pathway (Figure S25) was significantly influenced in both conditions, with Atp6v1b2 and Ppa2 upregulated in HN conditions and Ndufs4, Ndufv2, Ndufb11, Uqcrb, Cox5b, and Cox6c upregulated in HH conditions. However, several other proteins were downregulated in HN conditions, including Ndufs1, Ndufs2, Ndufs3, Ndufs4, Ndufs8, Ndufv2, Ndufa2, Ndufa5, Ndufa9, Ndufa10, Ndufb4, Ndufb5, Ndufb6, Ndufb8, Ndufb10, Ndufb11, Sdhb, Sdhc, Uqcrfs1, Cyc1, Uqcrc1, Uqcrc2, Uqcrb, Uqcr10, Cox4i1, Cox5a, Cox5b, Cox6c, and Cox7a2, and Cycs was upregulated in HH conditions. DNA replication was upregulated in HH conditions, including Pcna, Mcm2, Mcm3, Mcm5, Mcm6, and Mcm7.

## 3. Discussion

The global cost of treating type 2 diabetes mellitus and associated diseases is rising globally. Unlike other treatments, NMN has previously shown great potential in treating T2DM [11] with no observed adverse effects. It was previously proposed that NMN as a NAD^+^ precursor activates NAD^+^-dependent Sirt1 which, in turn, through TFAM and c-Myc transcription factors, induces mitochondrial biogenesis [12]. However, this seems to have a minor if not totally absent effect as trials in humans do not report the expected results [18]. Therefore, a discovery proteomics method was required to find hints about where glucose is going following NMN treatment. Proteins are stable, and they are the effectors and regulators of all biological functions of cells in living organisms [24]. A recent important advance in DIA data processing methods using artificial intelligence/neural networks has significantly improved proteome coverage and the limit of quantification [25].

As T2DM’s hallmark is impaired glucose uptake, we performed differential proteomics on the tissues responsible for most of the glucose consumption: the liver, muscle, adipose tissue, and the brain.

In our T2DM model involving cultured HepG2 cells, cardiolipin content (Figure A2A), as marker for mitochondrial mass, was significantly higher in both NMN-treated conditions (HN and HH); mitochondrial function was not significantly affected (Figure A2E), while membrane potential was lower (Figure A2F). Notably, in HH conditions, all the OXPHOS proteins were downregulated (Figure A5A and Figure S20), with the same pattern observed in HN conditions. Here, OXPHOS complex I component NDUFA3 (NADH: ubiquinone oxidoreductase subunit A3) and complex V subunits ATP5PD (ATP synthase peripheral stalk subunit d) and ATPAF2 (ATP synthase mitochondrial F1 complex assembly factor 2) were significantly repressed, while COX5a and NDUFb10 were significantly overexpressed by NMN treatment. Additionally, ATP6V1, a protein functioning as a channel that selectively allows protons to enter cell compartments like lysosomes, was overexpressed in HH conditions. In these hepatic cells, we also observed a downregulated proteasome pathway in HH conditions (Figure A4B and Figure A5), whereas, under the same conditions in myotubes, this pathway was upregulated (Figure A8). Interestingly, in HN and HH conditions, in HepG2 cells, ribosome components were overexpressed concomitant with repressed spliceosome components, except HSPA1B which was upregulated (Figure 8C and Figure A5A). This is more like muscle cells in HN conditions, and possibly, NMN somehow influences the splicing machinery (Figure A7C), resulting in abnormal proteins directed to chaperones for refolding or to proteasomes for recycling [26]. In HepG2 HN conditions, spliceosome components are downregulated along with processing in the endoplasmic reticulum, amino acid degradation, and fatty acid degradation (Figure A4A and Figure 8). Also, HSPA8 was repressed, as probably misfolded proteins were in a lower number and proteins synthesis in general was downregulated. It is worth mentioning that we found that, in hepatocytes, NMN treatment in HN conditions (Figure A4A) induced an upregulation of HLA-Cw, a leukocyte antigen (HLA) class I gene product, known to be involved in transplanted liver rejection [27]. This might raise concerns regarding NMN administration to liver transplant patients.

Our data also show upregulated TYMS, also known as thymidylate synthetase, an enzyme catalyzing the conversion of deoxy uridine monophosphate (dUMP) to deoxythymidine monophosphate (dTMP). It is involved in the regulation of DNA synthesis and cell proliferation. TYMS expression is upregulated in hepatocellular carcinoma, and its overexpression has been associated with poor prognosis and tumor progression [28]. SSBP1, also known as single-stranded DNA-binding protein 1, plays a critical role in DNA replication (Figure S22), recombination, and repair as well as the maintenance of genome stability [29]. KIAA0406, also known as TELO2 interacting protein 1 (TTI1), plays a critical role in the regulation of DNA damage response and cell cycle progression [30], and it was found significantly upregulated in NMN-treated HepG2 cells in HN conditions. Moreover, GRIK2, found upregulated in this condition, was previously reported to be involved in regenerating the liver after partial hepatectomy [31].

The study of differential protein expression in various tissues following NMN treatment revealed intriguing insights into the molecular mechanisms underlying its effects on glucose metabolism and cellular functions. Table 1 summarizes the key proteins identified and their functions in different tissues.

In vivo, our data showed similar downregulation of spliceosome components the in liver (Figure 3 and Figure S6), also observed in liver and muscle cells in vitro. Our data agree with those of Jiao et al. [32] that proved that in contrast to the m^7^G cap, which has a stabilizing role of mRNA, the 5′end NAD^+^ capping of eukaryotic RNA targets the rapid decay of mRNA in mammalian cells through the DXO de-capping enzyme. Probably, the energetic state of cells impacts NAD^+^ capping and mRNA turnover [33].

In mouse liver, ribosome components were also affected; in almost equal proportion, some components were upregulated while others were downregulated (Figure 2, Figure 3 and Figure S2), probably because Nolc1, a protein that localizes to the nucleolus and plays a role in ribosome biogenesis [34] (Figure S4), was downregulated. Downregulated Nolc1 and Clic4 are probably among the most important positive effects of NMN in the liver, as it was previously reported that Nolc1 contributes to the activation of hepatic stellate cells, which are key players in the development of liver fibrosis [35]. Both Nolc1 and Clic4 appear to be involved in the regulation of extracellular matrix production and may contribute to the progression of fibrosis [36]. Arachidonic acid metabolism, retinol, and fatty acid degradation were upregulated, probably due to increased NAD^+^ concentrations, which stimulated the desaturase activity of linoleic acid [37], the activities of retinol dehydrogenase and aldehyde dehydrogenases resulting in all-trans-retinoic acid, an agonist of α, β, γ receptors for retinoic acids [38], as well as the activity of L- β-hydroxy acyl CoA dehydrogenase. Steroid hormone biosynthesis was also affected, having some of the involved proteins upregulated and others downregulated (Figure 3 and Figure S1). Metabolism of xenobiotics by cytochrome P450 was upregulated, suggesting a better hepatic detoxification activity of the liver after NMN treatment (Figure S5).

Notably, Mt-Cyb (mitochondrially encoded cytochrome B protein) was overexpressed in the liver of NMN-treated mice (Figure 2), which could improve the electron carrier function of mitochondria. This effect was not observed in myotubes in vitro, probably suggesting that this mechanism occurs in other cells of muscle tissue but not in myocytes or it is dependent on a signaling pathway. Overexpression in the muscle tissue of NMN–treated mice was noticed for MPC1 (mitochondrial pyruvate carrier 1), NUP210L, Cep43, VNN1, CDC27, and TRAPPC3L, all found overexpressed in the muscle tissue of NMN-treated mice (Figure 4). Also, an interesting case is FTH1, a protein extremely important in iron storage and homeostasis [39], which is significantly overexpressed in liver tissue but also in cultured myotubes (Figure 2 and Figure A1).

Skeletal muscle plays a vital role in the regulation of post-prandial glucose levels. Following ingestion, approximately 80% of glucose is absorbed by skeletal muscle through a process known as insulin-dependent glucose uptake [40]. Both insulin-dependent and insulin-independent mechanisms are involved in the disposal of glucose by skeletal muscle. This process entails the delivery of glucose from the bloodstream to the muscle, its movement across the extracellular matrix towards the cell membrane, and uptake facilitated by specialized glucose transporters present either continuously on the cell membrane or translocated in response to insulin or exercise stimuli. Furthermore, intracellular glucose metabolism influences the creation of a glucose concentration gradient, thereby facilitating glucose transport within muscles [41].

**Figure 9:**
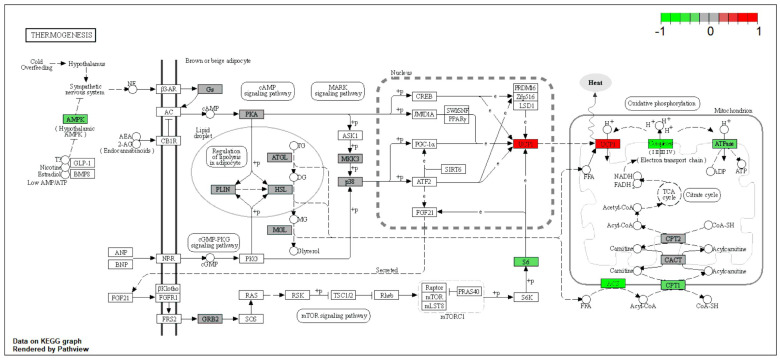
NMN treatment effects on the KEGG thermogenesis pathway in mouse muscle. The color of the boxes represents the log2 fold change of the protein abundances, represented for HFD + NMN group versus HFD group. Red: upregulated; green: downregulated; grey: no significant expression change.

In the muscle tissue, our data show that nothing happens in terms of reducing insulin resistance. While the expression of glucose receptor GLUT4 (Table 1), responsible for glucose internalization in muscle cells, is significantly reduced, the glycogen synthase (GS) enzyme is downregulated as well as most mitochondrial proteins in NMN-treated mice, which is contrary to the NMN-induced mitochondrial biogenesis hypothesis [12]. Our proteomic data strongly suggest that in the muscle tissue of HFD mice, NMN acts as a mild repressor for mitochondrial proteins. Skeletal muscle tissue is not made only by muscle cells, it might contain blood vessels, adipose cells, etc. Unexpectedly, we detected significant overexpression of UCP1 (Figure 9), which is a protein of the inner mitochondrial membrane of brown adipose tissue that acts as a regulated proton channel dissipating the proton gradient formed during the oxidation of NADH and FADH_2_ resulting from the metabolization of oxidable substrates. The energy of the proton gradient is not used for ATP synthesis but for heat generation [42]. This result confirms a previous study correlating NMN administration with thermogenesis [43].

To rule out the hypothesis that NMN could induce the expression of UCP1 in muscle cells, we used myotubes differentiated from C2C12 myoblasts in two different in vitro conditions, one with high-glucose and high-insulin growth medium during NMN treatment (HH) and the second with normoglycemic and normal-insulin-level medium (HN). Neither of the two conditions generated expression of UCP1, suggesting that, probably, in animals, NMN either stimulates the differentiation of preadipocytes infiltrated in skeletal muscle tissue into brown adipose cells or stimulates overexpression of UCP1 in existing brown adipose cells. These were not observed on histology images of the muscle tissue (Figure 5B) as 7 days of treatment was probably too short a duration for these brown adipose cells to grow or multiply to a visible extent. Thus, brown adipose cells were not observed in the skeletal muscle tissue. Furthermore, the examination of liver histology (Figure 3C) did not reveal the presence of brown adipose cells. It is known that BAT is present in most mammals, including mice and humans, in specific anatomical locations such as the interscapular region, as well as in proximity to organs such as kidneys, pancreas, and heart. In the present study, both WAT and BAT were observed in both conditions, specifically in perirenal adipose tissue (PRAT) and epicardial adipose tissue (EAT), respectively. Examination of histology images (Figure 6C) featuring PRAT and EAT reveals a coexistence of WAT and BAT, implying a transitional state from WAT to BAT, a phenomenon previously described by other researchers as “brite” or beige [44]. Notably, a clear morphological distinction exists between the two adipose tissue types. WAT exhibits conspicuous lipid accumulations manifesting as large intracytoplasmic vacuoles, in contrast to BAT, which displays numerous fine lipid vacuoles with a brownish hue, hence the designation. These findings further underscore the functional diversity and metabolic adaptability of these adipose depots, thereby enhancing our comprehension of adipose tissue biology and its implications for energy homeostasis.

**Figure 10:**
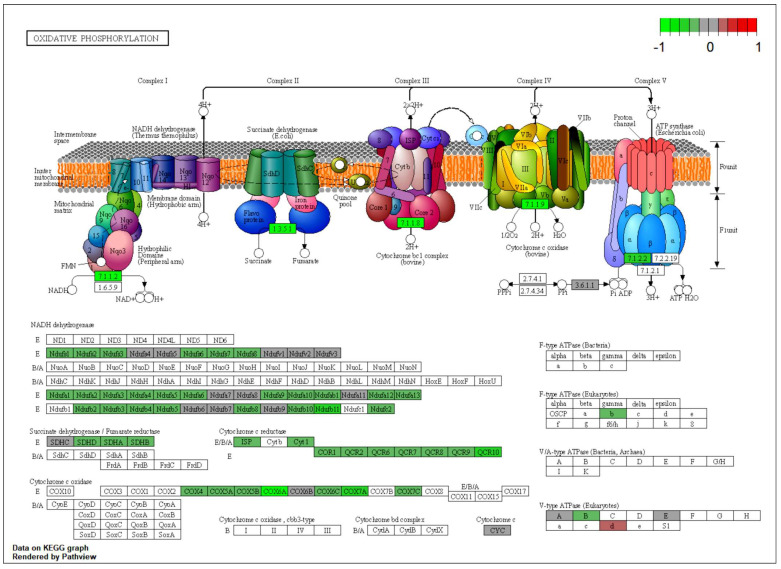
NMN treatment effects on the KEGG oxidative phosphorylation pathway in mouse muscle tissue. The color of the boxes represents the log2 fold change of the protein abundances, represented for HFD + NMN group versus HFD group. Red: upregulated; green: downregulated; grey: no significant expression change.

The evaluation of mitochondrial mass through cardiolipin content (Figure A3A) showed a slight decrease in both HN and HH NMN-treated cells. Also, mitochondrial function was reduced in both HN (*p* < 0.05) and HH (*p* < 0.0001) conditions while mitochondrial membrane potential was increased significantly in HN conditions (*p* < 0.05) and slightly but without statistical significance in HH conditions (Figure A3D–F). Mitochondrial reactive oxygen species production was increased by NMN treatment in HN conditions (*p* < 0.0001) and decreased in HH conditions (*p* < 0.01) (Figure A3G). The expression of OXPHOS proteins (Figure S25) in HN conditions was repressed by NMN treatment, the same effect being observed in vivo in similar conditions (Figure 10).

The decrease in mitochondrial function coupled with an increase in membrane potential and higher ROS production in muscle cells (Figure A3E–G) may be corelated with the decreased expression of Txnip (thioredoxin-interacting protein) (Figure A6A): Txnip is a protein that binds to and inhibits the activity of thioredoxin, playing a role in redox regulation [45]. It is a negative regulator of glucose uptake and its downregulation has been shown to increase glucose uptake in muscle cells [46] and increase reactive oxygen species (ROS) production [47]. It is highly conserved and plays a key role in regulating cellular redox balance and was shown to extend the lifespan of fruit flies [48]. Txnip was clearly observed as downregulated in our data along with improved glucose uptake but not in muscle tissue.

Protein synthesis was significantly upregulated in all NMN-treated muscle cells. Interestingly, the degradation of proteins within the proteasome pathway (Figure S26) was suppressed when high glucose and high insulin levels were maintained during treatment and was upregulated if normoglycemic medium was used during exposure. Also, TCA cycle proteins and fatty acid degradation were slightly downregulated in HN conditions and not significantly affected in HH conditions.

During this discovery proteomics study, in the treated HN myotubes, there was a downregulation of the spliceosome pathway (Figure S27) corelated with the upregulation of chaperones (Hspa8, Hspa11) and proteasome proteins in the treated HN myotubes. These findings suggest that NMN could lead to abnormal protein synthesis, activating chaperones and proteasomes for corrective actions (refolding or degradation). Also, NMN treatment leads to a higher NAD^+^ concentration, which might cause NAD^+^ capping followed by the rapid decay of mRNA through the DXO de-capping enzyme, previously described by Jiao et al. [32]. However, in muscle cells, NMN treatment in the high-glucose condition (HH) clearly shows downregulated proteasome and upregulated DNA replication and cell cycle pathways (Figure A8B), suggesting that cells‘ metabolism is on the growth and replication program. Overexpression of Znf827 (zinc finger protein 827) (Figure A6B) could suggest that NMN stimulates myoblasts’ differentiation of cultured cells in HH conditions. Treated cells in HN conditions showed upregulated purine metabolism and collagen synthesis, and, interestingly, a component of kinetochore, Zwilch (Zwilch kinetochore protein) was upregulated. Also, Hmg20a, a critical regulator of muscle differentiation and regeneration [49], was upregulated. There are a few other interesting protein expression differences in cultured myotubes shown on the heatmap (Figure A6), which might be useful as a starting point for other studies. However, so far, we rule out the mitochondrial biogenesis hypothesis as the main NMN effect in muscle.

**Figure 11:**
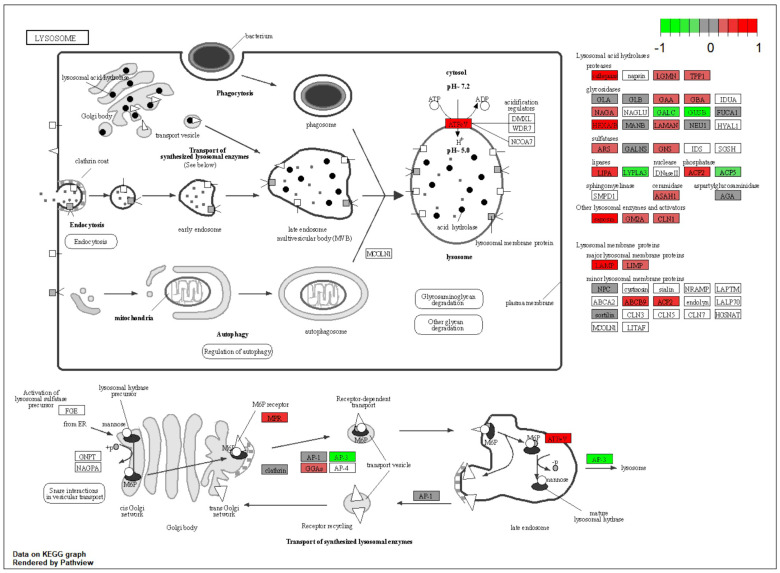
NMN treatment effects on the KEGG lysosome pathway in mouse adipose tissue. The color of the boxes represents the log2 fold change of the protein abundances, represented for HFD + NMN group versus HFD group. Red: upregulated; green: downregulated; grey: no significant expression change.

A tissue that consumes a considerable amount of glucose is the white adipose tissue, whose metabolism undergoes significant alterations in individuals with type 2 diabetes, leading to dysregulation of lipid storage, release, and adipokine secretion, contributing to the pathogenesis of the disease [50]. In NMN-treated mice, proteins from the mTOR cell growth pathway (Figure S14) are overexpressed, as well as proteins involved in amino acid and protein synthesis and degradation (Figure A1). All identified proteins involved in the lysosomal pathway (Figure 11) are strongly overexpressed and corelated with the overexpression of ATP6V1 required for the acidification of lysosomes, necessary for the activation of lysosomal lipases [51]. Interestingly, ATP6V1 is overexpressed in NMN myotubes in culture (Figure A7C). This hydrolyses the triglycerides stored in lipid droplets into free fatty acids and glycerol, which can then be used as an energy source. Adipose tissue is composed of adipocytes and a stromal vascular fraction (SVF) that includes preadipocytes, immune cells, and endothelial cells. Overexpression of tight junction proteins (Figure S15) facilitates vascular permeability [52]. Inflammation-related proteins (Cfd (complement factor D (adipsin)), Gm1088 (immunoglobulin kappa variable 5–48), Igk-V19-17 (immunoglobulin kappa variable 6–17)) are downregulated (Figure A1).

Retn (Resistin) is an adipokine hormone that is mainly secreted by adipose tissue and is involved in insulin resistance and inflammation [53]. Our data show reduced expression levels of Resistin corelated with upregulated Rida (reactive intermediate imine deaminase A homolog), which was previously shown as repressed in insulin resistance [54]. We consider that this is the way NMN works in T2DM and not as previously claimed by mitochondrial biogenesis. NMN treatment may have a beneficial effect on insulin sensitivity and inflammation by decreasing the expression level of Resistin and upregulating Rida. To our surprise, this is the only proteomics study which managed to relatively quantify Resistin by mass spectrometry.

The brain is also actively involved in the uptake and utilization of glucose, its primary energy source [55]. In NMN-treated HFD mice, brain tissue proteomic data (Figure 7 and Figure S16) revealed downregulated mitochondrial OXPHOS, with Atp5mk (ATP synthase membrane subunit DAPIT) and ATP5F1D (ATP synthase, H^+^ transporting, mitochondrial F1 complex, delta subunit) being the most repressed. Components of tight junctions were upregulated (Figure S17), similar to those of adipose tissue (Figure 6B and Figure 7B,C), while SNARE interactions in the vesicular transport pathway were downregulated as well as protein processing in the endoplasmic reticulum (Figure S18). Purkinje cell protein 4 (Pcp4) is primarily expressed in the cerebellum, specifically in Purkinje cells, which are a type of neuron involved in motor control and coordination [56], and was downregulated in the brain tissue of NMN-treated mice. During neural development, Numb (NUMB endocytic adaptor protein) is involved in regulating cell fate decisions by asymmetrically segregating into one daughter cell during cell division. This process, known as “Numb-mediated asymmetric cell division” leads to one daughter cell retaining stem cell properties, while the other becomes a neuron or glial cell [57]. In the context of neural development, repression of Numb (which is also our case) has been associated with an increase in symmetric cell division [58], where both daughter cells retain stem cell properties. Moreover, our studies revealed a downregulation of Ketohexokinase (KHK) in NMN-treated mice. This is an enzyme primarily involved in the metabolism of fructose and implicated in the pathogenesis of Alzheimer’s disease [59]. Previous studies have suggested that fructose may contribute to the development of Alzheimer’s disease by increasing the production of amyloid beta, involved in the formation of amyloid plaques in the brain [60]. By catalyzing the phosphorylation of fructose, KHK generates accumulation of glyceraldehyde, a metabolite that has been shown to increase the production of amyloid beta [61]. Decreased levels of KHK, as shown by our data, might have a positive NMN effect in the brain.

Also, solute carrier family 27 member 5 (SLC27A5) has been implicated in the regulation of the levels of very-long-chain fatty acids (VLCFAs) in the brain. SLC27A5 could play a role in the transport of VLCFAs into astrocytes, where they can be metabolized and incorporated into myelin. In addition, SLC27A5 has been shown to be upregulated in response to certain pathological conditions [62], and in our experiments, it is downregulated by NMN treatment.

The ABCB10 transporter is also downregulated by NMN exposure. ABCB10 has been shown to be highly expressed in neurons, and it has been suggested to play a role in the regulation of mitochondrial function in these cells [63]. It has been implicated in the response to oxidative stress, which is a common feature of many neurological disorders [64]. Its reduced level may be due to the reduced level of mitochondrial OXPHOS protein, which, in turn, may cause lower oxidative stress and lower the need for ABCB10, because of the higher level of NAD^+^. The same mechanism might explain the observed reduced levels of ELAV-like RNA-binding protein 4 (ELAVL4), with previous studies suggesting that ELAVL4 may also play a role in the regulation of the expression of genes involved in the response to oxidative stress [65]. Also, ATP-binding cassette, sub-family B (MDR/TAP), member 6 (ABCB6) was found upregulated and is a known transporter of hem (a known source of reactive oxygen species) out of the cells. This might be another protecting effect, as a mild repressed mitochondrion might require lower levels of hem to function. Cytosol-produced hem, not needed in mitochondria, is, thus, exported through the overexpressed ABCB6 carrier [66]. Also, albumin was overexpressed, which had been previously shown to have an antioxidant role in the brain, acting like a free radicals scavenger [67].

Wdfy1 (WD repeat and FYVE domain containing 1) is a large multidomain protein, found upregulated. It is involved in endosomal trafficking and it seems to play a role in the maturation and trafficking of endosomes as well as in autophagy [68]. Upregulated Kininogen 1 (Kng1) has also been shown to have some neuroprotective effects in the brain. It has been suggested that Kng1 may be involved in the regulation of cerebral blood flow and the protection of neurons from oxidative stress and inflammation [69]. Gnmt (glycine N-methyltransferase), found to be upregulated by NMN treatment, is an enzyme primarily involved in the metabolism of glycine as well as in the regulation of S-adenosylmethionine (SAM) levels. SAM is an important methyl donor in various biological processes, including the methylation of DNA and histones, which can affect gene expression [70]. Gnmt is involved in the catabolism of SAM, regulating SAM levels in the brain [71]. Decreased Gnmt expression has been observed with age and neurodegenerative diseases [72]. Also, TUBAL3, found upregulated in our experiments, is involved in the regulation of microtubule stability and organization, which are critical for proper neural progenitor cell division and migration [73]. Overexpression of Creld1 (cysteine-rich with EGF-like domains 1), known to be expressed in neural progenitor cells (NPCs) [74], suggests that NMN treatment might slightly stimulate the growing population of NPC cells.

Interestingly, HMGCS2, an enzyme involved in the synthesis of ketone bodies, which are important metabolic fuels for the brain during periods of fasting or low glucose availability [75], is upregulated in the brain of NMN-treated mice. This is interesting as these mice were not fasting nor exercising; thus, glucose should have been available as a brain energy source and there should have been no need for increased ketone bodies production. However, this might be an adaptation to reduced ATP production in mitochondria, also observed in our data and discussed above. Improved turnover of synaptic proteins might be induced by NMN treatment, as Ube2z [76] is significantly upregulated.

There are also several other upregulated proteins involved in synaptic plasticity, neuronal survival, neural development, and neuroinflammation: Prkcd (protein kinase C, delta), Syp (protein tyrosine phosphatase, non-receptor type 11) [77], Ly6h (lymphocyte antigen 6 complex, locus H), Coro2a (coronin, actin-binding protein 2A), Ppm1e (protein phosphatase 1E), Nptx1 (neuronal pentraxin 1), Cpne6 (copine 6), and Ildr2 (immunoglobulin-like domain containing receptor 2). Our data also show an NMN-induced overexpression of Ccdc136 (coiled-coil domain containing 136) in the brain. However, almost nothing is known about this protein; its structure was software predicted, but its function is unknown [78].

## 4. Materials and Methods

### 4.1. Animal Experiments and NMN Treatment

For HFD model experiments, male C57BL/6J mice of 20 weeks of age were obtained from the SPF Animal Facility. Mice were randomly divided into two groups, which consisted of 5 mice each. The animals were placed in open cages and provided with a standard laboratory diet and water *ad libitum*. All animals were grouped and housed in an environmentally controlled room with temperature between 19 °C and 23 °C and 30% to 70% relative humidity, with a 1 h light–dark cycle for an acclimation period of 7 days prior to the beginning of the experiment. After acclimation, both groups were fed with a high-fat diet (HFD) with 60% of the total calories from lard for a period of eight months to induce type 2 diabetes. The mice were considered diabetic when blood glucose levels at the two-hour time point after intraperitoneal glucose tolerance tests (IPGTTs) were higher than 200 mg/dL. After 7 days of NMN treatment (intraperitoneally administrated 500 mg NMN per kg body weight/day) for one group only (HFD + NMN), the mice were euthanized by cervical dislocation. All experimental procedures were executed in agreement with the regulations of the bioethical committee (authorization # 01/2020) of the Independent Research Association (Bucharest, Romania) and with the European legislation concerning experiments performed using live animals.

### 4.2. Serum Levels of Triglycerides and Cholesterol

Before euthanasia, blood samples were collected from the retro-orbital plexus for biochemical analysis of triglycerides and cholesterol using VetTest CHOL and VetTest TRIG strips with a clinical IDEXX VetTest Chemistry Analyzer System (IDEXX Laboratories, Inc., Westbrook, ME, USA).

### 4.3. Histochemical Observation

Immediately after euthanizing the mice, tissue samples from the skeletal muscle, liver, white adipose tissues, and brain were subsequently snap-frozen by immersion in liquid nitrogen vapors and stored at −80 °C for proteomic analyses and small pieces were fixed in 4% paraformaldehyde solution in phosphate buffer 0.05 M, pH 7.4, for a period of 48 h. After fixation, the specimens were embedded in paraffin wax. Sections (3–4 µm) were prepared and stained with hematoxylin and eosin (HE) for histological examination. Images were captured with an Olympus BX41 microscope. Images were generated using an Olympus DP25 Camera (Cell B software, Version 2.1).

### 4.4. Cell Culture, Differentiation, and NMN Treatment

The HepG2 cell line (ATCC HB-8065) was used as an in vitro model for the liver, while the C2C12 cell line (ATCC CRL-1772) was used as a model for muscle. The cells were grown in DMEM medium (Gibco, REF 31600-083) supplemented with 10% fetal bovine serum (FBS, Gibco), without addition of antibiotics, in a humidified atmosphere with 5% CO_2_ at 37 °C.

At 70% confluence, C2C12 myoblasts were differentiated by serum deprivation using DMEM medium and 2% horse serum (Horse Serum, Donor Herd, H1270, Sigma-Aldrich, Munich, Germany). After 3 days of differentiation, myotubes were grown for 3 days in hyperglycemic medium (DMEM + 30 mM glucose + 100 nM insulin), to induce insulin resistance. Since subsequent studies investigated the effects of NMN on mitochondrial activity and biogenesis, the culture medium was not supplemented with antibiotics (aminoglycoside antibiotics such as streptomycin have mitochondrial toxicity effects) to avoid affecting the aerobic metabolism of the cells. The culture medium was changed every 24 h. On the third day of culture, cells grown under hyperglycemic conditions were divided into two groups, so that during the NMN treatment, one part of the myotubes remained in hyperglycemic conditions, while the medium for the other part was changed to the specific normoglycemic condition (Figure A7A). Thus, two experimental conditions were obtained: cells grown in a hyperglycemic and hyperinsulinemic medium switched to a normoglycemic medium during the treatment (HN) and cells grown in a hyperglycemic medium before and during the treatment (HH). The cells were maintained with NMN treatment for 24 h. Control cells were grown similarly in parallel corresponding to each of the three conditions but with no NMN treatment. The same procedure was applied to the HepG2 cells (Figure 8A).

After treatment, the cells were harvested to assess the effects of NMN treatment. To determine the relevant concentration of NMN in the culture, initially, five concentrations were tested for cells’ viability and cytotoxicity: 50, 100, 500, 1000, and 5000 µM. For subsequent experiments, 100 µM NMN was selected as representative and consistent with other published studies. Cell cultures in all experiments were from passages 5 and 8 for the C2C12 line and passage 20 for the HepG2 line.

### 4.5. Flow Cytometry

After 24 h treatment with or without 100 µM NMN in HN and HH conditions, HepG2 cells and C2C12 myotubes were harvested and washed twice in cold PBS and incubated with different fluorescent dye solutions in culture media without phenol red and serum for 30 min at 37 °C in the dark. The fluorescent markers used were as follows: 10 nM NAO (sc-214487, Santa Cruz Biotechnology, Dallas, Texas, USA) for mitochondrial mass, 25 nM MitoView Red for mitochondrial function, 2 µg/mL JC-1 for mitochondrial membrane potential, 2.8 µg/mL DHR123 for mitochondrial ROS, and 100 ng/mL Nile Red (7726.1, Carl Roth, Karlsruhe, Germany) for neutral and polar lipids. After dye incubations, cells were washed with PBS and resuspended in 500 μL culture media without phenol red. The stained cells were analyzed on a Cytomics FC 500 flow cytometer (Beckman) (FL1/FL2/FL3/FL4/FL5) using Flowing software version 2.5.1. For each sample, three replicates of 15,000 events each were acquired.

### 4.6. Fluorescence Microscopy

For microscopic visualization of metabolically active mitochondria, mitochondrial membrane potential, and mitochondrial ROS, cells were cultured in 96-well plates—black/clear Sterile Imaging Plate (BD 353219, Falcon Corning, Glendale, Arizona, USA). After inducing insulin resistance and NMN treatment, the culture medium was aspirated from each well, followed by two washes with PBS. Subsequently, cells were separately incubated with 100 µL per well of MitoView Red (25 nM), JC-1 (2 µg/mL), and DHR123 (2.8 µg/mL) (GeneCopoeia, Rockville, MD, USA) diluted in serum-free culture medium at 37 °C in the dark. After 30 min, the staining solution was removed, and the cell surface was washed twice with PBS. Following staining, cells were maintained in phenol red-free culture medium (normoglycemic or hyperglycemic) and were visualized using an Olympus IX73 inverted fluorescence microscope.

### 4.7. Sample Preparation for Mass Spectrometry Analysis

After NMN treatment, tissue and cell samples were minced and homogenized in ice-cold lysis buffer (containing 8 M urea, 5 mM EDTA, 25 μg/mL spermine, 25 μg/mL spermidine, and 1 mM PMSF, in 50 mM Tris-HCl, pH 7.8) using a Retsch MM400 homogenizer with stainless-steel balls (⌀ 2 mm) for 5 min at ν = 30. The crude homogenate obtained after centrifugation was further centrifuged at 17,000× *g* for 10 min at 4 °C to isolate the protein fraction. The protein quantification was performed using the Bradford method and a bovine serum albumin 6-point (0.125–1.5 μg/μL) standard curve.

For proteolysis, a volume corresponding to 30 μg protein was diluted in 50 mM NH_4_HCO_3_ to a final volume of 500 μL, resulting in a urea concentration of 1.5 M, in low-binding 1.5 mL tubes. The samples were then incubated with 25 μL of 100 mM DTT in 100 mM NH_4_HCO_3_ for 45 min at 37 °C. To carry out alkylation, 26.25 μL of 300 mM IAA in 100 mM NH_4_HCO_3_ was added and incubated for 45 min at 37 °C in the absence of light. Trypsin digestion was performed using a trypsin solution with a concentration of 1 μg/μL (Trypsin Gold, V528A, Promega, Madison, WI, USA), and 0.6 μL of the trypsin solution was added to achieve a final ratio of 1:50. The mixture was left overnight at 37 °C with gentle shaking. The digestion process was stopped by adding 10 μL of 10% trifluoroacetic acid. The resulting peptides were purified using Spec plus C18 tips, speed-vacuum-dried, and reconstituted in 30 μL of a solution containing 2% acetonitrile and 0.1% formic acid. Finally, the samples were transferred to a clean autosampler vial with an insert for LC–MS/MS analysis.

### 4.8. Identification of Proteins by Liquid Chromatography–Mass Spectrometry Analysis

The peptide samples were subjected to LC–MS/MS analysis using an AB SCIEX TRIPLE TOF 5600+ mass spectrometer and separated with a NanoLC 425 system (Eksigent, Toronto, Canada). The setup included an analytical column (Eksigent 5C18-CL-120, 300 μM ID, 150 mm length) connected to DuoSpray ion source (AB Sciex, Toronto, Canada). A volume of 5 μL of the peptide samples was loaded using Solvent A (0.1% formic acid) and eluted with a gradient from 5% to 90% Solvent B (0.1% formic acid in acetonitrile) over 90 min at a flow rate of 5 μL/min, with a column temperature of 55 °C. Each sample was analyzed in triplicate.

Electrospray ionization in positive ion mode was used, with an ion spray voltage of 5500 V and a source temperature of 200 °C. The TRIPLE TOF 5600+ was operated in DIA SWATH-MS mode with 64 variable windows. The MS1 survey scan ranged from 400 to 1250 m/z, while the MS2 spectra were acquired in high-sensitivity mode from 100 to 2000 m/z. The accumulation time was set to 0.049 s, and the ion scan was sampled in 55 ms time windows in high-sensitivity mode, resulting in a cycle time of 3.5 s.

The mass spectrometry proteomics data were deposited into the ProteomeXchange Consortium via the PRIDE [79] partner repository with the dataset identifier PXD043257. Reviewer account: Username: reviewer_pxd043257@ebi.ac.uk; Password: M0KNo3iI.

### 4.9. Data Analysis

Protein identification from DIA data was carried out in a label-free and library-free manner using DIA-NN ver. 1.8.1 [25]. The raw spectra were searched against the fasta file containing the complete mouse reference proteome (UniProt, UP000000589, November 2022, 55,275 entries) for mouse tissue samples and C2C12 myotubes and against the complete human reference proteome (UniProt, UP000005640, November 2022, 82 492 entries) for HepG2 cells, with a precursor m/z range of 400 to 1250, and trypsin was chosen as the digestion enzyme. The data were searched with MBR enabled and robust LC was employed as the quantification strategy, with a false discovery rate (FDR) set at 0.01. C carbamidomethylation and Ox(M) modifications were considered during the search. Retention time-dependent normalization was applied, and quantitation was performed using the MaxLFQ [80] algorithm in DIA-NN.

Statistical and differential downstream analysis was conducted using the locally installed PolySTest version 1.3 (release) [20], which can be downloaded from the following link: https://bitbucket.org/veitveit/polystest/src/master/ (accessed on 10 January 2023). The analysis utilized as input the unique gene matrix *tsv file obtained from DIA-NN. For significance determination, a *p* value threshold of 0.05 was applied, along with a log_2_ fold change (log_2_FC) threshold of −0.2–0.2 or −0.3–0.3 or −0.5–0.5 to identify significantly downregulated or upregulated proteins. In PolySTest, the Limma test was used for statistical significance analysis, as it is well suited for gene or protein expression data and handles well the missing values in some replicates. Following the statistical analysis, data visualization, including heatmaps and expression profiles, was generated.

Each gene identified through DIA-NN was converted and mapped to its corresponding protein object using the “org.Mm.eg.db” and “org.Hs.eg.db” packages in Bioconductor [81,82]. The proteomic data were analyzed and graphically plotted primarily using the R Studio platform (version 4.2.2).

For the differential pathway expression analysis (PEA), the list of proteins with their respective log2 fold change and statistical significance was exported from PolyStest and imported into R, for further analysis using PathfindR version 1.6.4 [83]. PathfindR utilizes a protein–protein interaction network (PIN) analysis approach with PIN data for *Homo sapiens*. For mouse tissues and C2C12 myotubes, the code was optimized to take PIN data for *Mus musculus* obtained from STRING (https://stringdb-static.org/download/protein.links.v11.5/ (accessed on 10 January 2023)), taxon id 10090. The output from PathfindR is a table that displays enriched pathways identified from the protein list, including fold enrichment values, the lowest and highest *p* values generated from each pathway analysis iteration, and the upregulated and downregulated proteins associated with each pathway [83]. Additionally, we generated an enrichment chart and term–gene graph for the top 20 and top 10 KEGG pathways, respectively, sorted by lowest *p* value.

For data integration and visualization of the main biological processes, we utilized the KEGG pathway database [21,22,23] (https://www.genome.jp/kegg/pathway.html (accessed on 10 January 2023)) and employed the Pathview R package version 1.38.0 [84].

## 5. Conclusions

Overall, improved glucose uptake observed after NMN treatment seems to be caused mainly by effects in the adipose tissue, namely Resistin downregulation and increased protein synthesis and degradation, fatty acid degradation, and lysosome protein upregulation (most notably upregulation of the ATP6V1 proton pump), along with mTOR cell proliferation signaling in white adipose tissue; and differentiation of preadipocytes to brown adipose cells and/or overexpression of thermogenic UCP1. A series of other effects in an organ-type-dependent manner were also observed. Among these, it worth mentioning that spliceosome downregulation corelated with upregulated chaperones, proteasomes, and ribosomes in liver and muscle cells, resulting in slightly impaired and energy-inefficient protein synthesis machinery, increased production of ketone bodies through Hmgcs2, downregulation of some mitochondrial OXPHOS components and the TCA cycle in the brain, and overexpression of proteins involved in the metabolism of xenobiotics in the liver. Notably, our data strongly suggest that NMN is not acting through mitochondrial biogenesis, the opposite seems to be the case, having a mild repressing effect on mitochondria, inducing known positive effects like those observed in animals during fasting. As a discovery study, this work aimed to provide a clear picture of the NMN treatment effect in T2DM and a starting point for further investigations to further elucidate the mechanisms responsible for the most interesting of the observed effects.

## Figures and Tables

**Table 1:** Proteins significantly altered in tissues by NMN treatment.

Tissue	Protein Name/Pathway	Function	Expression Change
Liver	Nolc1	Ribosome biogenesis	Downregulated
Liver	Clic4	Regulation of extracellular matrix production	Downregulated
Liver	Mt-Cyb	Improves the electron carrier function of mitochondria	Upregulated
Liver	FTH1	Iron storage and homeostasis	Upregulated
Liver			
Muscle	NUP210L	Transport of macromolecules between the nucleus and cytoplasm	Upregulated
Muscle	Cep43	Regulation of microtubule dynamics and spindle formation during mitosis	Upregulated
Muscle	VNN1	Regulation of oxidative stress and inflammation	Upregulated
Muscle	CDC27	Regulation of the cell cycle	Upregulated
Muscle	TRAPPC3L	transport of proteins between the endoplasmic reticulum (ER) and Golgi apparatus	Upregulated
Muscle	GLUT4	Glucose internalization in muscle cells	Downregulated
Muscle	UCP1	Thermogenesis	Upregulated
Adipose	mTOR pathway	Cell growth	Upregulated
Adipose	Lysosomal pathway	Lipid metabolism	Upregulated
Adipose	Resistin	Adipokine involved in insulin resistance	Downregulated
Brain	OXPHOS proteins	Mitochondrial function	Downregulated
Brain	Tight Junctions	Cell membrane structure	Upregulated
Brain	ABCB10	Regulation of mitochondrial function in neurons	Downregulated
Brain	Synaptic plasticity proteins	Neuronal survival and development	Upregulated
Brain	HMGCS2	Ketone bodies synthesis	Upregulated
Brain	Ube2z	Improved turnover of synaptic proteins	Upregulated

Nolc1—nucleolar and coiled-body phosphoprotein 1; Clic4—chloride intracellular channel 4; Mt-Cyb—mitochondrially encoded cytochrome B protein; FTH1—ferritin heavy polypeptide 1; NUP210L—nucleoporin 210-like; CEP43—centrosomal protein 43; VNN1—vanin 1; CDC27—cell division cycle 27; TRAPPC3L –trafficking protein particle complex 3-like; GLUT4—glucose transporter type 4; UCP1—uncoupling protein 1; ABCB10—ATP-binding cassette sub-family B member 10; HMGCS2—3-hydroxy-3-methylglutaryl-coenzyme A synthase 2; Ube2z—ubiquitin-conjugating enzyme E2Z.

## Data Availability

The data presented in this study are available on request from the corresponding author. The mass spectrometry proteomics data have been deposited into the ProteomeXchange Consortium via the PRIDE [103] partner repository with the dataset identifier PXD043257.

## Supplementary Materials

The following supporting information can be downloaded at: https://www.mdpi.com/article/10.3390/ijms25052594/s1.
